# Community-Driven Water Quality Assessment Following the 2023 Maui Wildfires: Insights into Post-Fire Drinking Water Contamination and Resilient Disaster Response

**DOI:** 10.1021/acsestwater.5c00896

**Published:** 2026-01-05

**Authors:** Liza A. McLatchy, Andrew J. Whelton, Kexin C. Rong, Kellie D. P. Cole, Julynn I’i, Christopher K. Shuler

**Affiliations:** † Water Resources Research Center, 371665 University of Hawai’i at Ma̅noa, Honolulu, Hawaii 96822, United States; ‡ Lyles School of Civil and Construction Engineering, Division of Environmental and Ecological Engineering, 311308 Purdue University, West Lafayette, Indiana 47907, United States

**Keywords:** wildfire, drinking water, Maui, community response, volatile organic compounds (VOCs)

## Abstract

The August 2023 wildfires in Maui, Hawai’i, damaged or destroyed more than 2200 structures and displaced thousands of people. Residents in the towns of Kula and Lahaina were put under do-not-use drinking-water advisories due to the potential for volatile organic compound (VOC) formation within or leaching from the water distribution system following heat or smoke exposure. This study documents how researchers and community members united to initiate a home tap water sampling and water-quality outreach program in response to the need for information during and after the crisis. The majority of samples were collected in the three months after the wildfire and were screened for 78 VOCs, many of which were fire-related compounds. In total, 395 raw-tap water samples and 191 filtered water samples were analyzed. Fourteen chemicals were detected; however, very few exceedances of drinking water exposure limits were found. A key success of the program was the employment of affected community members as sampling staff, which fostered trust, improved participation, and enhanced communication. The findings offer insights into the impacts of urban wildfires on municipal water systems and the important role university-community collaboration can play in disaster response.

## Graphical abstract

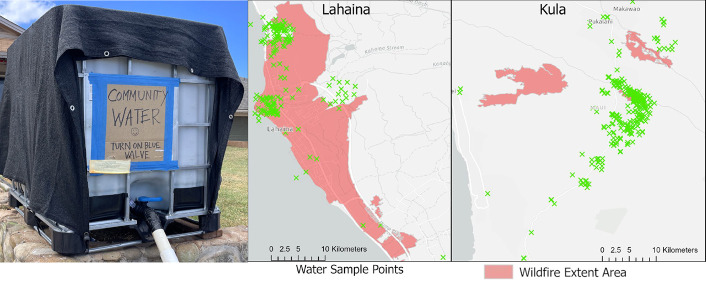

## 1 Introduction

Wildfires have long been recognized as destructive to both the environment and to critical infrastructure. In particular, recent urban wildfires have introduced new public health challenges related to drinking water safety. Following California’s Tubbs and Paradise fires in 2017 and 2018, investigators identified widespread chemical contamination in drinking water distribution systems due to heat and smoke exposure.^13,14,15^ Since 2018, more than ten urban-wildfires have contaminated municipal water systems in California, Colorado, New Mexico, Oregon, and now Hawai’i, posing health risks to residents and causing significant economic disruptions to recovering communities.^20^

In August 2023, a historic extreme wind event ignited four wildfires on Maui, Hawai’i, two of which entered the towns of Lahaina and Kula, respectively (Figure 1). These fires impacted densely populated areas, destroying or damaging over 2200 structures, resulting in substantial loss of life, and displacing more than 20,000 residents, many of whom remain displaced nearly two years later. The Lahaina fire stands as the deadliest U.S. wildfire in the past century. In addition to the immediate devastation, returning residents faced ongoing uncertainty about the safety of their drinking water.^19^ As observed in other urban wildfires, concerns over contamination from volatile organic compounds (VOCs) in drinking water led the county water department to issue “do-not-use” advisories.^19^ This hazard, increasingly recognized in fire-impacted water systems, arises from the thermal degradation of plastic pipes and other synthetic plumbing components, which can release VOCs into water when exposed to extreme heat.^6,9,10,13^ Moreover, depressurization or open pipes caused by fire damage may cause smoke (condensate) and hazardous vapors or solid fire debris (e.g., ash) to be drawn into household plumbing, introducing further contamination that can spread to the system and other homes.^8^ Coupled with pressure losses due to leaks and firefighting demand, these factors made it highly likely that VOC-contaminated water from burned structures had spread throughout the distribution system.^6^

**Figure 1:**
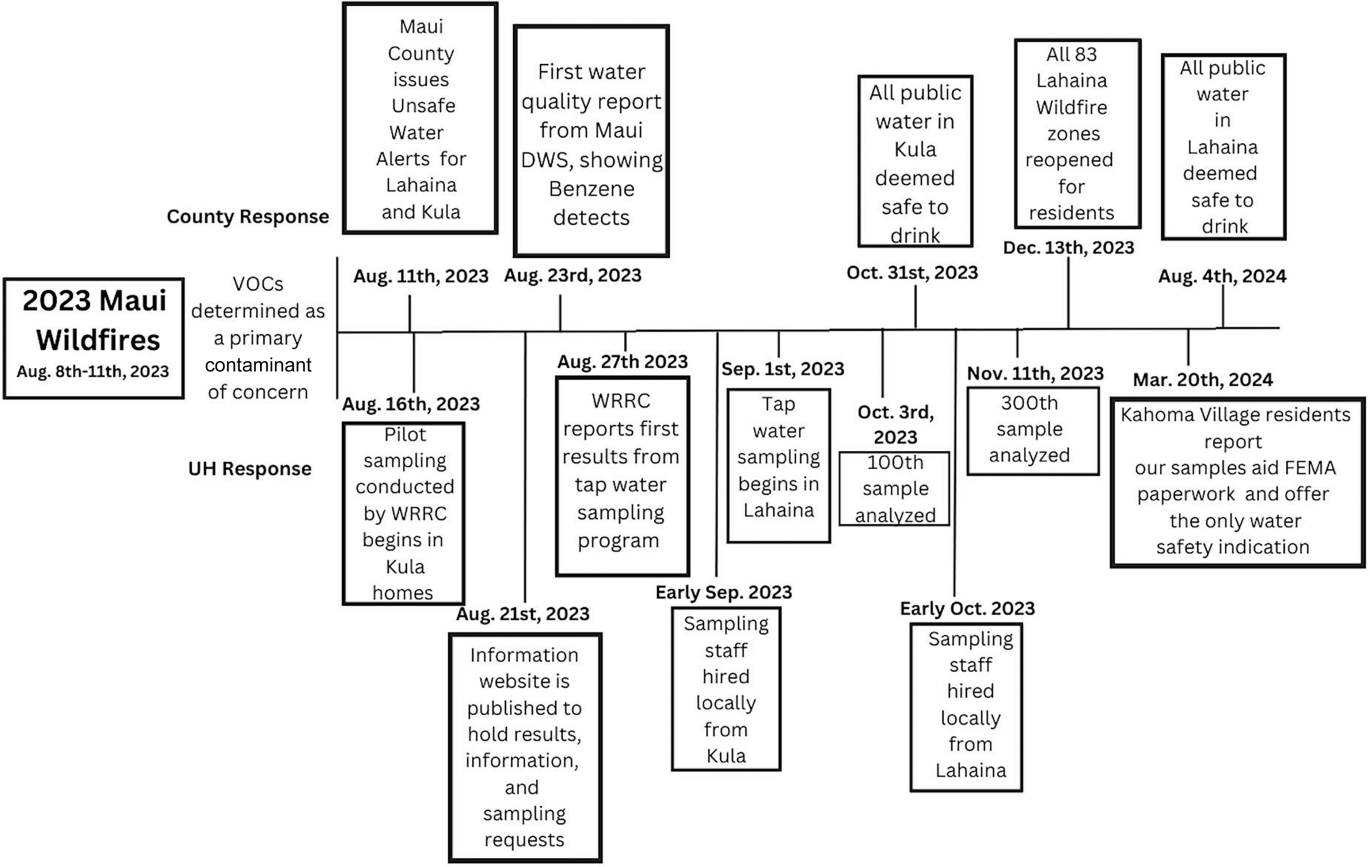
Event timeline and overview of the drinking water response from both Maui County and the University of Hawai’i.

As in other disaster scenarios, once evacuation orders were lifted, homeowners began seeking guidance and support for drinking water testing.^12,15^ However, most local government and regulatory agencies remained overwhelmed by immediate response efforts and were unable to provide the level of direct engagement residents sought.^20^ This pattern is common following major disasters, where urgent county water operational priorities, such as stopping leaks and restoring firefighting capacity, often take precedence over efforts to communicate water quality risks or offer public guidance. In response, some residents turned to commercially available water testing kits and attempted to treat their drinking water with household filters.^20^

Despite the great need for information about drinking water safety in wildfire-affected areas, few research-driven efforts have addressed community-level concerns or tested water from household plumbing located on the household side of the water meter. A prior study showed that after the 2018 Camp Fire, the lack of safe drinking water was associated with increased levels of anxiety, stress, and depression in impacted residents.^12^ Another study conducted drinking water sampling inside homes and found chemical contamination remained nine months after the disaster.^15^ Similarly, after the 2021 Marshall Fire in Colorado, chemical contamination persisted for months, including in service lines connected to both standing and destroyed homes.^5^ After the Maui wildfires, residents urgently sought to determine whether their tap water posed a health risk.

In this study, we document the rapid design, deployment, and implementation of a community-driven home drinking water sampling program. Within 1 week of the fires, our research group began collecting tap water samples from residents. This program was coproduced with Kula and Lahaina area residents to enhance trust and access. To address critical information gaps, we rapidly designed and launched a community-driven home drinking water sampling program through the University of Hawai’i Water Resources Research Center (WRRC). Sampling began in Kula, where early field observations and test results informed protocols later applied in other affected areas. Our findings aim to inform future policy development and emergency response planning.

## 2 Methods

### 2.1 Overview

To meet urgent community needs and support our research objectives, our approach was to offer free drinking water testing to households impacted by the Lahaina and Kula Fires, with participation facilitated through community outreach and an online form. Flyers with a QR code linked to a sampling request form were distributed during county-sponsored wildfire response community meetings, community centers, resilience hubs, and other local gathering places. The request form sought contact information and also prompted residents to describe concerns with their water. Using that information, residents were contacted through text, phone calls, or email to arrange appointments for water sampling. Geographic Information Systems (GIS) were used to map addresses and property locations to help our sampling team prioritize requests. Water samples were collected from properties with the following priority: (1) inside the wildfire-impacted zone, (2) properties adjacent to the wildfire-impacted zone, and (3) properties further away. This home water sampling program was designed to focus specifically on water collected “after the meter,” representing the privately owned portion of the distribution system. This approach differs from most postfire investigations that sample primarily on the utility side of the system. At the same time, Maui Department of Water Supply (DWS) conducted its own sampling program within the public distribution system. DWS informed us that they also tested water at the treatment plant sources out of an abundance of caution and detected no fire-related contamination. Because source waters and treatment facilities were unaffected by the fires, our sampling design did not include testing at the treatment plants and instead focused on household tap water to assess community exposure and private plumbing conditions.

Our initial team included one laboratory analyst and several staff and faculty from the University of Hawai’i campuses on Maui and O’ahu who focused on field sampling. As the number of property sampling requests quickly grew, we recognized the need to strengthen community engagement and logistical capacity. In response, we hired four community members from Lahaina and Kula to support sampling and outreach activities, recruiting them through local advertising and by disseminating the job announcement through professional, collaborative, and community networks. Concurrently, as the water sampling program was being launched, our team also published a website, the *Maui Post-Fire Community Drinking-Water Information Hub*, designed to centralize critical water-related information for affected residents. The site went live on August 21st, 2023, to act as a useful resource for updates, frequently asked questions, contacts, and data from our sampling efforts.^16^

### 2.2 Onsite Water Sample Collection

At each home or sampling location, we asked the property owner or resident where they preferred the sample to be collected. Kitchen and bathroom sinks were the most common collection points, although we also collected samples from outdoor spigots, particularly when residents were not present during the visit. We asked each participant about the presence of any water filtration systems, both at the tap and whole-house filters. Samples consisted of unfiltered tap water, which is most representative of water within the distribution system. In cases where residents requested analysis of filtered water, we adapted by collecting two samples: one of unfiltered tap water and one of the filtered water. While this dual-sample approach was not part of the original study design, we incorporated it frequently to support participant concerns and enable a field assessment of home-filter performance.

Water samples for volatile organic compound (VOC) analysis were collected using 40 mL amber glass vials equipped with Teflon-coated septum caps. Each vial was predosed with 25 mg of ascorbic acid (Thermo Scientific, Inc., >99%) to neutralize residual chlorine. Before sampling, we allowed water to flow at a steady trickle for one to 2 min to flush the tap and avoid aeration or spraying. We gently filled each vial by letting water run down the inner side of the bottle to minimize bubbles and splashing. To preserve sample integrity, we filled each vial fully to eliminate headspace without overflowing, ensuring the ascorbic acid remained inside. To prevent damage during transport, we wrapped bottles in bubble wrap and placed them on ice in a cooler. At the end of each sampling day, we preserved the samples by adding 250 μL of 1:1 HCl (Fisher Chemical, ACS+). All samples were refrigerated at 4 °C until shipment to the laboratory for analysis. Weekly, samples were overnight-shipped directly to the University of Hawai’i WRRC Environmental Chemistry Lab on Oahu for analysis. The holding time for samples preserved with HCl was ideally limited to 14 days, though due to the shipping process, this hold time was exceeded in about 18% of our samples by an average of 1 week. Throughout storage and transport, samples were kept refrigerated, and every effort was made to store them separately from organic solvents to prevent cross-contamination.

We developed a rigorous logistics pipeline to ensure that community-based staff were consistent in collection methods and documentation. This included specific sample labeling and naming conventions, use of fillable online forms for metadata, and paper copies of wet-signed consent forms to ensure residents were aware of our planned use of the data. We also tracked all communications with residents via a team email account to make sure results were delivered back via email or text promptly. Before sampling, we asked each participant to sign a consent form, which we scanned and archived daily. If a participant had not signed a consent form, we required its submission before releasing their results. Each sample was documented in an internal-shared spreadsheet, accessed only by our staff, to ensure chain of custody information was recorded and securely stored.

### 2.3 Water Quality Analysis and Comparisons

We screened water samples for 78 VOCs using purge and trap (Teledyne Tekmar Atomx XYZ purge and trap concentrator using a #9 trap) and Gas Chromatography-Mass Spectrometry (GC-MS) (Thermo Scientific Trace 1300 GC, ISQ_LT MS). We selected these compounds based on our lab’s capacity with chemicals in EPA method 524.2 and their prior detection in drinking water systems following wildfires^13,15,20^ ).Commercially available certified reference materials were purchased from Restek (30601, 30006, 30020, 30074, and 30073) and stored below −20 °C in glass vials fitted with Mininert valves. Calibration standards were prepared in deionized water. A combined internal and surrogate standard mix (10 μg/mL) was prepared in purge and trap grade methanol (Fisher Chemical). A 5 mL aliquot of each sample or standard was transferred to a fritted sparge tube along with 10 uL of the internal and surrogate standard mix. Each sample or standard was purged with nitrogen for 11 min at 40 mL/min. A dry purge was performed at 100 mL/min for 0.5 min to remove excess water. The trap was then heated to 250 °C to desorb the analytes and transfer them to the GC. Desorb time was set to 0.5 min. Separation was carried out on a Restek Rxi-624Sil MS column (20 m × 0.18 mm × 1.0 μm) with helium carrier gas. The inlet was operated in split mode with a split ratio of 50 and a temperature of 200 °C. Column flow was set to 0.5 mL/min. The oven was programmed at 35 °C for 3 min, increased at 14 °C/min until 110 °C, then increased at 25 °C/min until 220 °C, and held for 17 min. The MS transfer line was held at 230 °C, and the ion source temperature was 300 °C. A *m*/*z* range of 35 to 260 was scanned with a dwell time of 0.15s. Calibration, internal, and surrogate standards were prepared in deionized water or purge and trap grade methanol (Fisher Chemical), from commercially available certified reference materials (Restek 30601, 30006, 30020, 30074, and 30073). Stock standards were stored below −20 °C in glass vials fitted with Mininert valves. A discussion of laboratory methods validation can be found in the Supporting Information (S1).

Our primary objective was to help residents better understand the quality of the drinking water in their homes. If any sample was found to exceed a *Safe Drinking Water Act* (SDWA) maximum contaminant level (MCL), regulatory authorities, specifically the Hawai’i Department of Health, were alerted. All postprocessed laboratory data were made publicly available through a GitHub repository (10.5281/zenodo.17365218), which also includes sample metadata and the code scripts used to process the raw laboratory data.

Health limits for each VOC in water were sourced from two separate regulatory entities: (1) the U.S. Environmental Protection Agency (EPA) under the *SDWA,* ^17^ which establishes MCLs, MCLGs for drinking water across the U.S., and (2) the Minnesota Department of Health (MDH), which provides Health Risk Limits (HRLs) for Minnesota’s drinking water.^11^ The MDH HRLs were selected because they are widely recognized as one of the most comprehensive and health-protective state-level standards in the United States, derived through detailed toxicological reviews that consider both short-term and chronic exposure pathways, including chemicals not currently regulated under EPA drinking water standards.

### 2.4 Distribution of Results to Residents

Water testing results were typically available within 2 weeks after sample submission; however, in some cases, processing and delivery took up to 6 weeks. Once results were available, we postprocessed the sample data using an open-source code (https://github.com/cshuler/VOC_Processing_Maui/blob/main/Scripts/VOC_Sample_Results_Round4.ipynb) to display results in a customized, user-friendly color-coded report seen in Figure 3. The reports included anonymized addresses (showing only the street, removing the house number) and date stamps, ensuring they could be useful for further data analysis or public sharing of data. Residents were typically supplied with their sample results via email, unless another method was either preferred or email was unavailable. Results were also posted in an online repository once identifying information was removed, as well as comparisons to relevant limits. Our team reported back to residents with several resources, including the color-coded report mentioned above, and a supplementary portable document format (PDF) file to explain how to read sample reports, where health limit information was sourced, and how to interpret the water quality data, shown in Figure 2.

**Figure 2:**
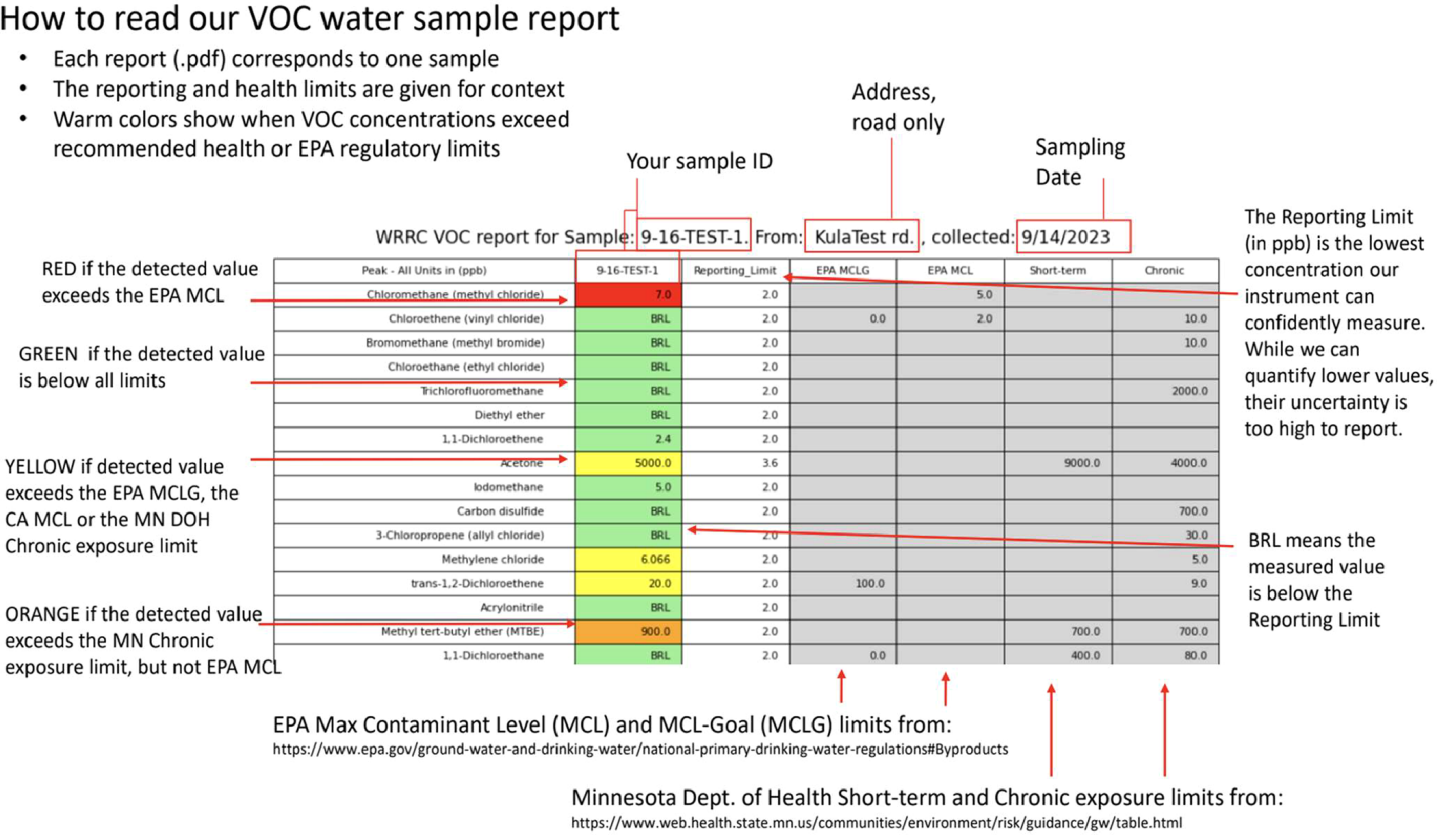
The document provided to the household included an explanation of the information that it contained in the context of relevant health and regulatory water quality metrics. This a direct screenshot from the report that was shared with homeowners. Note: in the beginning of the program to ensure completeness, multiple health agencies were being considered for comparison to the program’s samples. It was ultimately decided that adding more health limits than the two selected was unnecessary. We changed which limits we were reporting but “CA” remained in the generated report.

**Figure 3:**
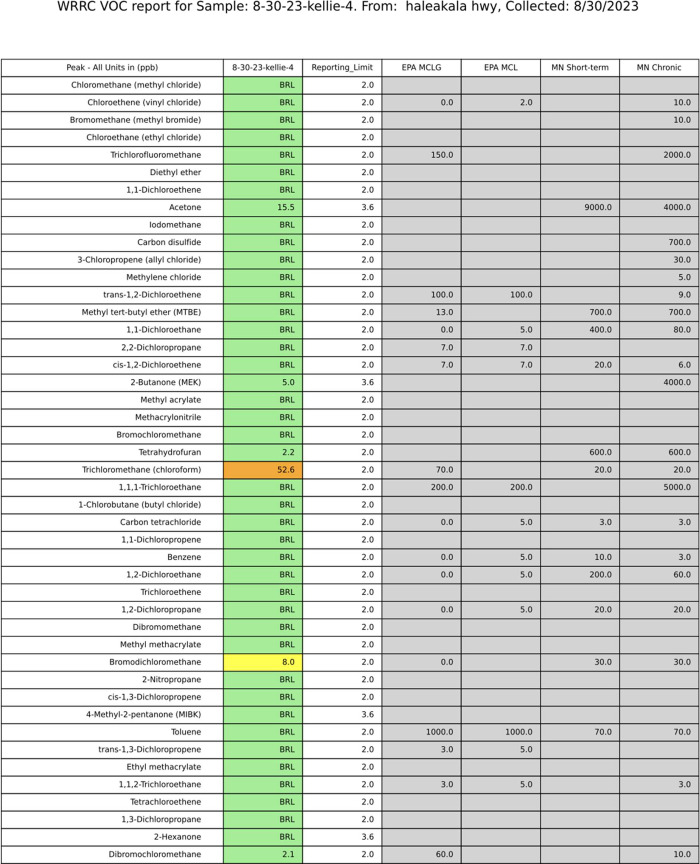
Example participant report that was sent to residents. Personal identifying information was removed from the publicly available reports. Note: this report’s length was reduced to fit the page.

In response to the community’s need for accessible information, we also published a website, the Maui Post-Fire Community Water Info Hub, hosted on the University of Hawai’i WRRC server.^16^ This aspect of our program aimed to empower residents with knowledge, offering them clarity and support as they navigated water safety concerns in the aftermath of the wildfire. The “Info Hub” was created to help communicate water quality data and address concerns by including information and resources related to postfire contamination. The site featured a request form for water sampling, along with tables and figures that compared detected compounds in Kula and Lahaina water samples to the same health-based limits used in participant reports (Figure 3). To support data interpretation, we included a Frequently Asked Questions (FAQ) section covering topics such as water safety, sampling methods, and how to understand lab results. To further support residents, the “Info Hub” provided links to information on health advisories, water quality standards, and other state and federal initiatives on postfire water quality. The site listed WRRC points of contact, allowing community members to reach out with additional questions or requests for data interpretation. Additional screenshots and an archived version of the Maui Post-Fire Community Drinking-Water Information Hub are provided in the Supporting Information (Section S6).

## 3 Results

### 3.1 Sampling Requests and VOC Detection Results

In the first three months following the disaster, we received 379 water sampling requests. By October 2024, 14 months after the disaster, we had more than 450 total requests. In total, we collected and analyzed 586 unique water samples, excluding lab or method blanks (Figure 4). The number of samples exceeded the number of requests because, at several properties, we collected both unfiltered and filtered water samples for comparison, including 395 raw-tap water samples (unfiltered) and 191 filtered-tap water samples. We received sampling requests across the island from households in the burn zone, adjacent to the burn zone, and far outside of the impacted areas on Maui (Figure 5). Of the 78 chemicals tested, only 14 chemicals were detected at concentrations above the method reporting limits. It should be noted that several of the volatile organic compounds (VOCs) measured in this study, including total trihalomethanes (TTHMs), are classified by the U.S. EPA as regulated disinfection byproducts (DBPs) formed when chlorine reacts with organic matter during water treatment and distribution, thus, distinguishing between operationally driven DBP variability and potential fire-related effects is an important consideration discussed further in Section S4 of the Supporting Information.

**Figure 4:**
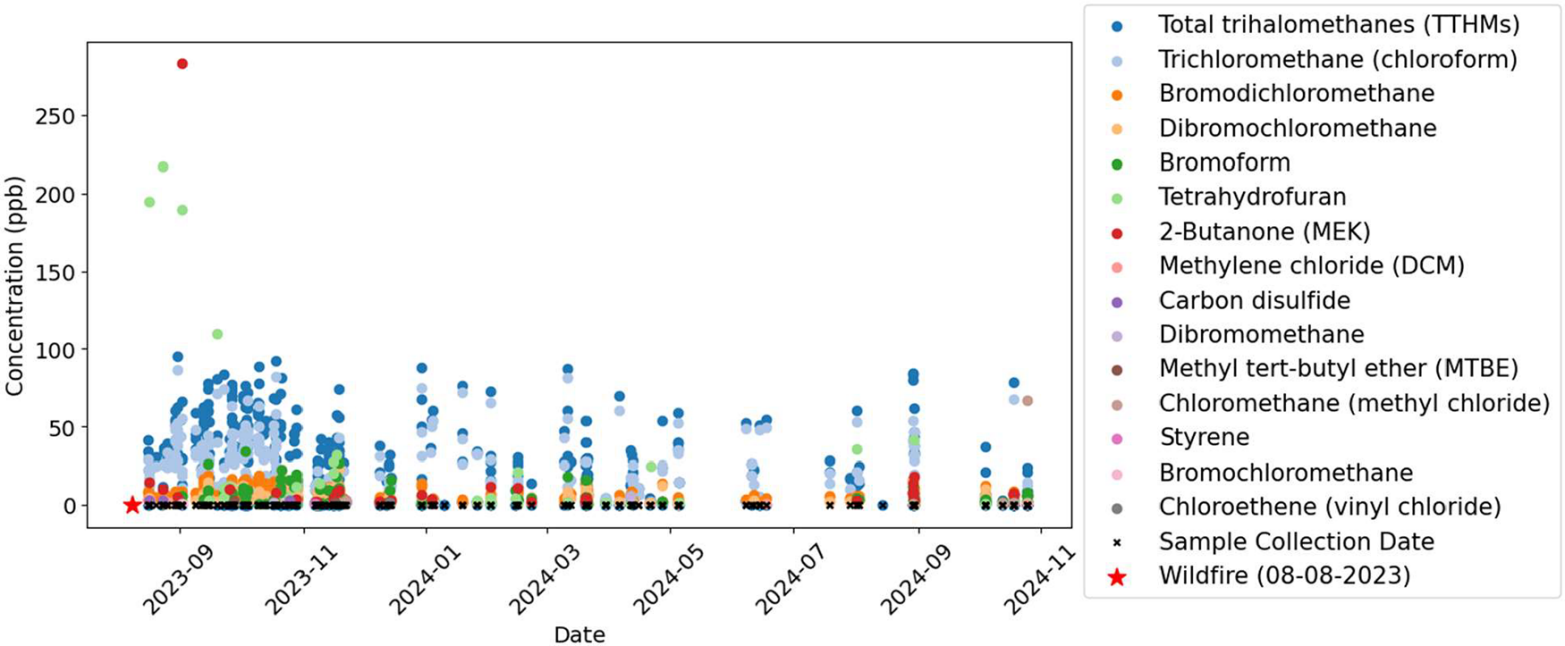
Time-series plot of raw-tap water samples (*n* = 390) and detected chemicals reported (concentrations above the reporting limits) throughout the study, corresponding with sample collection dates. Note: TTHMs is the accumulation of the trihalomethane chemicals: bromodichloromethane, bromoform, dibromochloromethane, and trichloromethane (chloroform).

**Figure 5:**
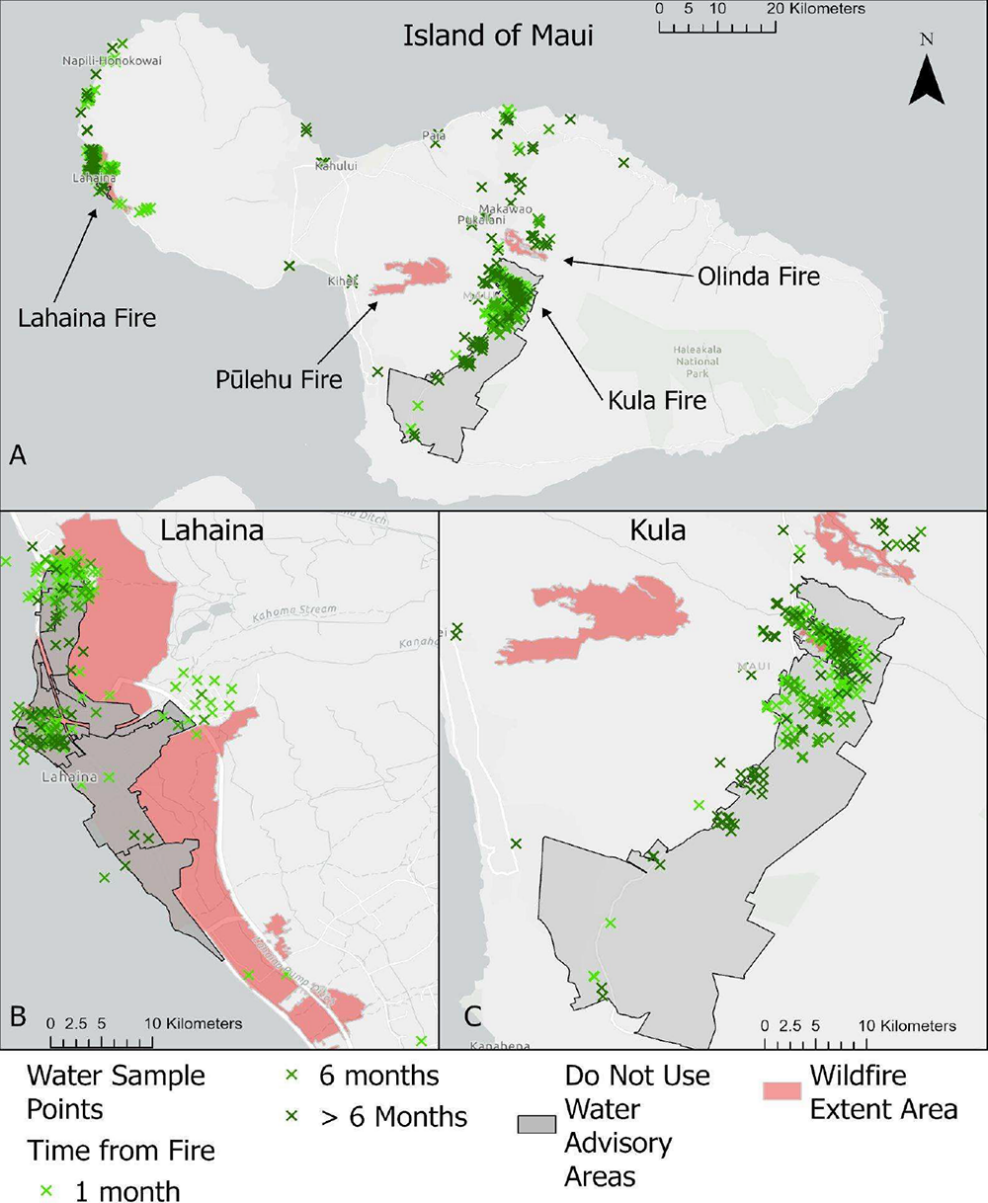
Households in and outside the burn zones for the County of Maui (A), for the Lahaina Fire (B), Kula Fire (C), Pu̅lehu Fire (C), and Olinda Fire (C) that requested drinking water sampling and analysis. Red shaded areas represent the wildfire-burn zones, and gray shading indicates the portions of the municipal water service areas placed under do-not-use advisories (though they do not indicate the full extent of County water service areas).

We first detected styrene, chloroethane (vinyl chloride), and methyl *tert*-butyl ether (MTBE) between November and December 2023, shortly after the fires. Tetrahydrofuran and carbon disulfide showed their highest concentrations within the first month following the disaster but continued to be detected throughout the study period. Analysis of total trihalomethanes (TTHMs) showed a decreasing trend between March and July 2024, followed by another increase in late August and September 2024. The temporal fluctuations in TTHMs appear consistent with operational changes in the water system following the fires. Communications with the water utility indicated that elevated TTHM concentrations were anticipated early in the recovery period due to increased chlorination during system restoration, with subsequent variability likely reflecting ongoing repair or maintenance activities. Tetrahydrofuran and carbon disulfide had their highest concentrations within the first month following the fire, but were still detected throughout the study. It is not uncommon to see certain wildfire-related chemicals detected long-after the fire, as chemicals can persist in the contaminated pipes.^20^

Most of our observed chemical detections occurred in water samples collected soon after the wildfires, primarily from households closest to the burn zone. Of the chemicals identified, all have been previously found in contaminated drinking water distribution systems after wildfires elsewhere.^5,6,13,15,20^ The maximum concentrations of VOCs we recorded were: styrene (1.23 μg/L), tetrahydrofuran (THF) (216 μg/L), vinyl chloride (VC) (1.67 μg/L, exceeding EPA MCLG), bromochloromethane (BCM) (1.04 μg/L), methylene chloride (2.92 μg/L), methyl-*tert*-butyl ether (MTBE) (3.01 μg/L), bromoform (33.9 μg/L, exceeding EPA MCLG), dibromochloromethane (DBCM) (23.0 μg/L), bromodichloromethane (BDCM) (19.3 μg/L), trichloromethane (TCM, chloroform (86.3 μg/L, exceeding EPA MCLG/MNDP Short-term and Chronic), chloromethane (methyl chloride) (66.9 μg/L), dibromomethane (6.17 μg/L), carbon disulfide (3.15 μg/L), 2-Butanone (ethyl-methyl ketone, MEK) (282 μg/L), and total trihalomethanes (95.2 μg/L, exceeding EPA MCL).

During detailed examination of the VOC data, we identified several unexpected patterns that prompted additional investigation into potential analytical artifacts. Acetone is recognized as a wildfire-related chemical and has been found postfire in other water distribution systems.^2^ However, acetone was detected in nearly all of our water samples, an unexpected finding that warranted further investigation. To assess potential sources of acetone, we conducted controlled tests in the laboratory using deionized and tap water with and without ascorbic acid and hydrochloric acid preservatives, over short (<1 day) and long (14 day) holding periods. Our results indicated that the industry-standard sample handling methods we used inadvertently generated acetone, most likely due to high concentrations of dissolved organic carbon reacting with the ascorbic acid preservative over longer hold times. This artifact affected only acetone results and did not appear to alter the concentrations of other VOCs. As a result, we excluded acetone from our reported findings. Similarly, our analysis of hold times showed that samples with longer storage durations had higher concentrations of trihalomethanes, reflecting ongoing DBP formation during refrigerated storage. This pattern was consistent with known reactions between chlorine residuals and natural organic matter, particularly in early samples from Kula where both hold times and organic carbon levels were elevated. Additional explanations of our analysis of both of these patterns as well as further analysis of sample preservation methods is explored in the Supporting Information (S3).

To determine whether any detected VOC posed a health risk to the household, we compiled information from previous wildfire testing, regulatory health limits, and the expertise of trusted researchers experienced in the subject. Table 1 below details the chemicals detected within tap water samples and compares their detections with the limits listed (if applicable).

**Table 1:** Results for Detected Chemicals in Tap Water Samples, Their Number of Detects, and if Those Detections Violated Any Known Limits, for All Raw Water (Unfiltered) Samples (*n* = 390)^a^

	health limits (μg/L)	detects (#)				
chemical (MRL in μg/L)	EPA MCL/MCLG:MDH C/ST	total detects	above EPA MCL	above EPA MCLG	above MDH chronic	above MDH short-term
trichloromethane (chloroform) (1 μg/L)	–/70:20/20	342	–	5	148	148
bromodichloromethane (1 μg/L)	–/0:30/30	249	–	249	0	0
dibromochloromethane (1 μg/L)	–/60:10/–	212	–	0	30	–
bromoform (1 μg/L)	–/0:–/–	95	–	95	–	–
tetrahydrofuran(THF) (1 μg/L)	–/–:600/600	35	–	–	0	0
2-butanone (MEK) (2 μg/L)	–/–:4000/–	33	–	–	0	–
methylene chloride (1 μg/L)	–/–:5/–	12	–	–	0	–
carbon disulfide (1 μg/L)	–/–:700/–	4	–	–	0	–
dibromomethane (1 μg/L)	–/–:–/–	5	–	–	–	–
methyl *tert*-butyl ether (MTBE) v	–/–:700/700	2	–	–	0	0
chloromethane (methyl chloride) (1 μg/L)	–/5:–/–	3	0	–	–	–
bromochloromethane (1 μg/L)	–/–:–/–	1	–	–	–	–
chloroethene (vinyl chloride) (1 μg/L)	2/0:–/–	1	0	1	0	–
total trihalomethanes (TTHMs) (4 μg/L)	80/–:–/–	367	9	–	–	–

a Not all chemicals have known limits; therefore, a “–” denotes the absence of a published health limit. All health limits are reported in μg/L, and the detections are reported in the number of samples meeting the criteria.

Our results revealed the widespread presence of chlorinated compounds, specifically Trihalomethanes (THMs), in municipal tap water and some filtered water samples. These include chlorinated compounds commonly found as disinfectant byproducts (chloroform, bromodichloromethane, and dibromochloromethane). We interpret these as most likely originating from standard water disinfection practices, although such compounds can also result from wildfire-related contamination; for example, all of these chemicals were detected in fire-damaged (nondisinfected) PVC drinking water distribution systems in other fires.^2^ Maui County describes its pipes as lead, nonlead, and galvanized, though some customer service lines at burned water meters were found to be PVC.^19^

### 3.2 Filter Efficacy for Low Levels of VOCs

While not an initial objective of the study, chemical analysis of water collected upstream and downstream of in-home water filters revealed significant variation in filter performance, though the majority of filters were fairly effective at reducing VOC levels to some extent. We commonly encountered two types of filters: (1) point of use (POU) filters (e.g., under-sink filters), (2) point of entry (POE) filters (e.g., whole house filtration systems). For each household that requested a filtered water sample, raw (unfiltered) water was first collected, then water downstream of the filter was collected in a separate bottle and labeled as a separate sample.

Among the 149 paired filtered-unfiltered samples we collected, 88 of them showed marked reductions in VOC’s, often in the 80–100% efficacy range. Note that filter efficacy was calculated as the average percent reduction across all detected VOCs per sample pair rather than based on summed concentrations. A total of 56 pairs showed limited filter efficacy, meaning we saw no or only minimal (<±10%) difference between filtered and raw water samples. Interestingly, 5 pairs showed increased VOC concentrations after filtering exceeding an addition of more than 10% from their filter-pair’s initial tap sample concentrations for one or more VOCs. These somewhat surprising outcomes from the latter two categories may result from several factors, including expired or saturated filters, filter housings that retain water and increase chemical contact time, filters not rated for VOC removal, or in numerous cases, we hypothesize that we detected adhesives from the recent installation of filtration systems. These cases typically displayed low-level detections of solvents (e.g., THF, MEK) that are commonly used in PVC glues.^3^ Conversations with residents confirmed a rise in home filtration installations after the wildfire, particularly whole-house systems that often require new piping and fittings, and our results suggest that some VOCs may leach from recently installed piping.

Unfortunately, only a subset of the filtered tap water samples included additional metadata on filter type. When available, this information allowed us to quantify the efficacy of each filtration system and allowed our team to provide residents with constructive guidance when explaining results. The most commonly used filter types were reverse osmosis (RO) systems and carbon filtration, and are compared in Figure 6.

**Figure 6:**
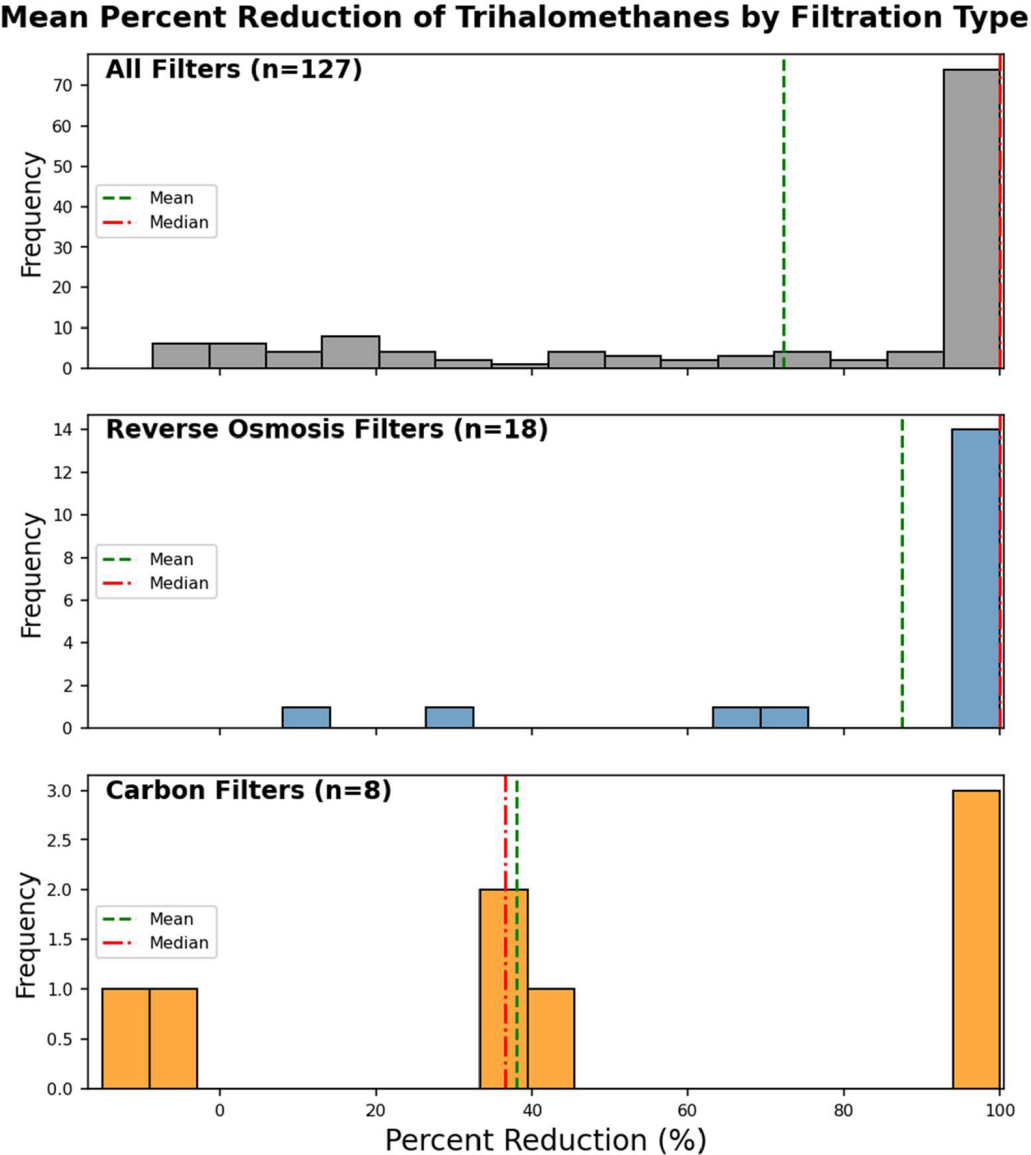
Distribution of average percent reduction of trihalomethanes by filtration type. The most detected chemical group was TTHMs; therefore, the average percent reduction per pair of the chemicals within that group is displayed. Samples with filtration mechanisms that added chemical concentrations were excluded outside a noise threshold (10% added). The top panel represents all filtered pairs, the middle panel displays pairs with a known filtration mechanism of reverse osmosis for the filtered sample, and the bottom panel displays pairs with a known filtration mechanism of a carbon filter for the filtered sample.

The testing of filtered samples was adapted into the sampling procedure at the direct request of residents. Residents expressed to us that they wanted to compare their filtered water sample results with their tap water sample results to determine the efficacy of their filter for the detected chemicals. A key finding of this analysis was that roughly 9% of household filters were not effective, either because filter elements needed to be changed or because the filters themselves were ineffective at removing VOCs. The paired data with chemical concentrations can be found on the project’s Github Repository (https://github.com/cshuler/VOC_Processing_Maui, 10.5281/zenodo.17365218). While we did not collect comprehensive data on the time of last element replacement or filter types, novel insights into a very applied issue could be gained from expanding this type of study in future research. Overall, our ability to meet residents’ requests for adaptations to our sampling program empowered residents to make informed decisions about their drinking water, including their filtration systems.

### 3.3 Field Experiences and Community Engagement

A novel aspect of our sampling program was its community-led codevelopment, undertaken during the fast-moving context of a natural disaster and accompanying crisis of trust in public institutions. Community-based sampling staff personally reached out to community members, conducted site visits, and addressed any questions they may have had. They coordinated schedules, tracked participant responses, and navigated logistical challenges unique to each neighborhood. Both physical and intangible challenges were evident, including road closures, downed trees, and unsafe conditions, such as poor air quality, active smoke from smoldering structures and brush, unstable ground on cliffs where trees and soil had burned, interactions with persons who had experienced trauma, and distrust from community members.

The high number of water sampling requests was driven by strong community awareness of the free testing program and by the fact that many residents remained in their homes within Unsafe Water Alert zones. We observed the peak in sampling demand during the first few months following the fires, with 216 requests in August 2023 alone. Requests gradually declined, reaching their lowest point in December 2023, before rising again in early 2024, likely due to residents returning home to properties in Lahaina, as reflected in participant testimonials. The temporal distribution of sampling requests is shown in Figure 7. To meet the increasing demand and improve community engagement, we expanded our community-based sampling team in early September 2023 by hiring two additional sampling staff members from Lahaina.

**Figure 7:**
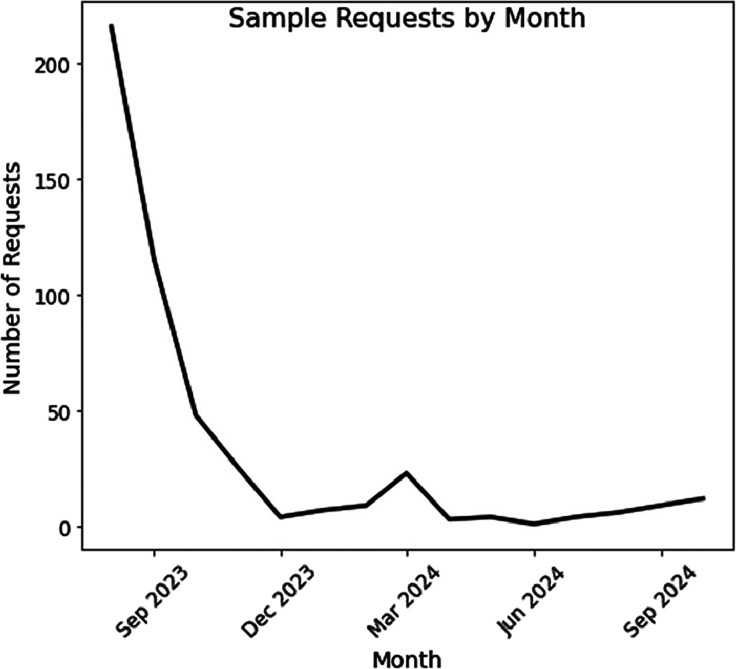
Volume of cumulative monthly sample requests throughout the sampling program. Note that data reflects formal requests from our online form, but does not reflect requests directed to sampling staff by word of mouth, which made up a significant fraction of requests toward the end of the program.

As part of the sampling request form, residents were prompted to describe their specific drinking water concerns. Out of 478 sampling requests received, 400 of the sample forms included concerns about the safety of their water. Common themes included general uncertainty about the safety of drinking water, potential exposure to contaminants produced by urban wildfires, health risks associated with unknown chemicals, doubts about the effectiveness of home filtration systems, and how proximity to burn areas might affect contamination risk. Residents also expressed frustration over limited communication from governmental agencies and concern about the cost and accessibility of private water testing kits. This information helped us inform sampling and programmatic design, develop the participant reports, update our online resources, and improve our ability to address residents’ anxiety around drinking water safety.

We received informal feedback from residents on the clarity of report formats and the delivery of test results. Many residents expressed appreciation for the accessibility of the reports, particularly the color-coded health limit key. Common questions concerned water safety, filtration options, and the need for retesting. Team members responded individually to these inquiries to ensure residents understood their results. These interactions also helped our team refine how we communicated technical information, though no formal evaluation of feedback was conducted. Our team reviewed and discussed feedback from residents internally and made sure that all participants’ questions were answered. Team members shared the responsibility of responding to residents’ concerns, confirming that all questions were answered as effectively as possible, and using a system of double-checks to ensure that all of the questions received were responded to individually over email, text, or in person, in a timely manner. About a year after launching the program, we reached out to all residents over email to ask for additional feedback, including improvements, through another online form. Responses included words of appreciation and satisfaction, as well as suggestions on improving our reporting and communication.

To further simplify our results for the community, we conducted additional data analysis by location, focusing on the areas of Kula and Lahaina. The reports, Figures S5.3 and S5.4 in the Supporting Information were created to be posted on the information hub with the purpose of effectively communicating concerning results to community members, based on their area of interest. The color coding also follows the same method as homeowner reports to maintain consistency.

## 4 Discussion

### 4.1 Detections, Absences, and Communicating Complex Water Quality Data

Our study detected fire-related chemicals in few drinking water samples, with only a handful of detections surpassing comparable health limits. Our study focusing on tap water samples within standing homes is one of the largest-scale postwildfire home tap water sampling programs to date, targeting water quality within plumbing systems past the water meter. The results of all our tap water samples showed that only one chemical class, TTHMs, violated an MCL set by the EPA under the SDWA. TTHMs are a well-recognized combination of disinfectant byproducts, but they have also been detected in other impacted water systems postfire when disinfectants are not used.^2^ Given the disruption to Maui’s water infrastructure, the temporary rise in TTHMs was anticipated, and the Department of Water Supply (DWS) communicated that levels were expected to decrease over time. Utility monitoring from 2018–2025, analyzed in the Supporting Information (S4), shows seasonally elevated TTHMs in both systems; while no utility samples exceeded the EPA MCL (80 μg/L), ∼20% of Kula detections (36/183) and ∼22% of Lahaina detections (28/127) were above their 2022 system-specific maxima (44 and 62 μg/L), and MDH guidance values for chloroform and dibromochloromethane were intermittently exceeded. Through our outreach and conversations, we found that many residents were unaware that these chemicals were present in their drinking water before the fire, and that chlorinated compounds were common in treated water. To make this information available, we updated our FAQ section with information providing clarity to residents.

The presence of TTHMs underscored the challenges of conveying complex water quality information to the public. Additional complexities, such as trace detections, reporting limits, and the public’s unfamiliarity with certain chemicals, further complicated communication efforts. Laboratory contamination also introduced challenges, as illustrated by our experience with acetone. Investigating the potential sources of this unexpected detection required time and added uncertainty to our results, contributing to residents’ confusion and complicating data interpretation. Additionally, while our emphasis on household responsiveness was critical for building trust and engagement, we recognize that this flexibility came at the expense of being able to implement a carefully designed sampling plan that would have maximized comparability and statistical power, a trade-off we highlight as important for future postdisaster study design. Maintaining direct lines of communication with residents allowed for more in-depth discussions, helping to clarify these concerns.

### 4.2 Success of University-Led, Community-Integrated Rapid Disaster Response

This study allows us to analyze the unique role of a university partnership with community members in a disaster response. In this case, our academic-community collaboration helped address critical knowledge gaps at a time when government agencies faced capacity limitations. Such challenges are not uncommon, as it is well-known that specific disaster and humanitarian aid authorities face barriers to a timely response.^4^ The success of our rapid response was influenced by several factors. Extensive local community connections (two coauthors’ homes were under an Unsafe Water Alert issued for Kula and Lahaina) allowed our team to tailor our program to residents’ needs. A key factor in the response was our decision to act immediately, due to our physical presence on Maui, before any funding was secured. By mobilizing early and responding to community needs as they emerged, we were able to collect time-sensitive data in the immediate aftermath of the fires. This rapid approach demonstrated a successful model that ultimately attracted funding from national research agencies and philanthropic community foundations.

University-community relationships keep communities informed and encourage them to take greater personal responsibility, thus motivating them to stay prepared for future risks in their area.^1^ This was evident throughout our study, with affected community members joining our team, residents providing feedback for the improvement of the program, and the large-scale interest in receiving water quality testing. The interdisciplinary nature of higher education is naturally equipped to adapt to the diverse needs of communities.^7^ For example, while the *SDWA* regulates only 97 chemicals out of the 60,000 in use,^18^ we were able to apply additional expertise to include various health limits to support residents in understanding water quality contamination. Similarly, university-community collaborations benefit all stakeholders, as a strong community directly contributes to the university’s well-being.^7^ When requesting feedback from residents, we asked where else the university could support the community to continue this collaborative relationship.

### 4.3 Feedback for Improvement of Future Collaborations

Our program received feedback throughout the course of the study from participating residents. While overall satisfaction was high, residents highlighted several opportunities for refining the program, which we analyzed to better understand how our and other similar programs can improve. A recurring theme was confusion around varying laboratory reporting limits. Residents requested clearer explanations of what these limits represent, why they vary, and how to interpret values reported below or near detection thresholds. Residents also expressed a desire for more accessible scientific communication. Specifically, they recommended including actionable next steps based on their results and simplifying the language used in report summaries. These suggestions emphasize the importance of designing outreach materials that provide clarity and minimize jargon.

Crises that follow a natural disaster cause uncertainty to rise, and the need for efficient communication between the community and responding organizations is even more crucial (Odimayomi et al.). While we maintained ongoing dialogue with residents throughout the process, we did not conduct structured interviews or collect demographic data, such as education level or prior knowledge of water quality risks. This limits our ability to assess how effectively we communicated risk and highlights the need for future programs to incorporate more intentional tools for evaluating public understanding.

These lessons allow us to suggest recommendations for future studies under similar circumstances: (1) codevelop communication materials with community input, (2) provide clear, tiered explanations of water testing results, (3) define regulatory versus nonregulatory thresholds explicitly, and (4) include mechanisms to evaluate participant comprehension and information needs throughout the program lifecycle.

## 5 Conclusions

Urban wildfires have introduced new public health challenges in recent years, especially as the wildland-urban interface (WUI) continues to expand.^20^ Drinking water system contamination of VOCs as a result of these urban fires is a recent discovery, with researchers and water system operators struggling to implement rapid, widespread testing. Communities and municipalities are trying to better understand the effect of urban wildfires on drinking water contamination in order to protect households and businesses from contamination and to accurately plan recovery efforts. Initiated by the University of Hawaii WRRC, a community-driven, postfire, home drinking water sampling program was implemented within less than 1 week following the 2023 Maui fires to detect potential contamination. This is the largest home tap water sampling program following an urban wildfire to date.^15^

From August 2023 to October 2024, our sample results showed the presence of fire-related VOCs, but only TTHMs (a combination of chemicals, detailed in the results) exceeded the federal MCL. Results suggest that residents within our study experienced very limited to no drinking water contamination.

This study highlights the success of a community-led disaster response program and the potential for this framework to be implemented in disaster scenarios. Creating a direct relationship between the university and community allows for a direct communication pipeline to be opened and utilized for response and recovery functions. This pipeline allowed community members to voice concerns and university researchers to provide solutions. In this format, university researchers are able to respond directly to community members, providing the ability to alleviate anxieties regarding public health safety and increase trust in the solutions fostered through this communication pipeline. Feedback provided throughout the course of the study allows us to make suggestions for future community programs. This study exemplifies how universities can serve as useful institutions during disasters, particularly when government resources may be limited or delayed.

## Supplementary Material



## Notes

Instrument calibration and quality control procedures; comparison of method reporting limits with health-based standards; evaluation of sample preservation effects, hold times, and artifactual acetone formation; statistical analyses of VOC exceedances; historical disinfection-byproduct data for Lahaina and Kula systems; advisory-area overlap, exceedance locations, and health-limit comparisons; and archived visual outputs from the Maui Post-Fire Community Drinking-Water Information Hub (PDF)
