# Supplementary material for: Community-Driven Water Quality Assessment Following the 2023 Maui Wildfires: Insights into Post-Fire Drinking Water Contamination and Resilient Disaster Response
Source: ACS ES T Water. 2026 Jan 5;6(2):793–804. doi: 10.1021/acsestwater.5c00896 (PMC12910593; doi:10.1021/acsestwater.5c00896)
Supplement: Supplementary file 1 [file ew5c00896_si_001.pdf]

Supplemental Information

Community-Driven Water Quality Assessment Following the 2023 Maui Wildfires: Insights into Post-Fire Drinking Water Contamination and Resilient Disaster Response

Liza A. McLatchy, Andrew J. Whelton, Kexin C. Rong, Kellie D. P. Cole, Julynn I'i, Christopher Shuler*

1. Liza A. McLatchy, [lmclatch@hawaii.edu] Water Resources Research Center, University of Hawai'i at Mānoa

2. Andrew J. Whelton, [awhelton@purdue.edu] Lyles School of Civil and Construction Engineering, Division of Environmental and Ecological Engineering, 550 Stadium Mall Drive, 3145 Hampton Hall, Purdue University, West Lafayette, Indiana, USA

3. Kexin Rong, [krong@hawaii.edu] Water Resources Research Center, University of Hawai'i at Mānoa

4. Kellie D.P. Cole, [kpenman@hawaii.edu] Water Resources Research Center, University of Hawai'i at Mānoa

5. Julynn I'i, [iijulynn@hawaii.edu] Water Resources Research Center, University of Hawai'i at Mānoa

*** Corresponding author:** Christopher K. Shuler, [cshuler@hawaii.edu] Water Resources Research Center, University of Hawai'i at Mānoa

Section S1. Instrument Calibration and Quality Control Procedures

We analyzed continuing calibration verification (CCV) standards at the start of each analytical batch. Analysis proceeded if (a) more than 80% of target analyte responses were within 30% of the true value, (b) internal standard responses were between 50% to 200% of initial calibration values, and (c) retention times of internal standards were within 30s of initial calibration values. If the system did not meet all CCV criteria, a recalibration was performed before proceeding with sample analysis. A blank was analyzed after each CCV to remove carryover contamination. A second blank was analyzed to confirm that target analyte concentrations in the blank are less than half of the method reporting limit before proceeding with sample analysis. A CCV standard was also analyzed at the end of each analytical batch. The mean % recovery and relative standard deviation of CCV standards (n=74) for analytes detected during the project are shown in Table 1.

Table S1: Mean recovery and relative standard deviation (RSD) of continuing calibration verification (CCV) standards, with the Method Reporting Limit (MRL) documented after each chemical in µg/. Only target analytes detected are included in the analysis.

Chemical (MRL in µg/L)	Mean Recovery (%)	RSD (%)
2-Butanone (MEK) (2 µg/L)	95.6	9.55
Bromochloromethane (1 µg/L)	96.5	11
Bromodichloromethane (1 µg/L)	90.8	10
Bromoform (1 µg/L)	90.8	11.8
Carbon disulfide (1 µg/L)	93.4	16.6
Chloroethene (vinyl chloride) (1 µg/L)	93.6	16.9
Chloromethane (methyl chloride) (1 µg/L)	96.8	19.5
Dibromochloromethane (1 µg/L)	91.3	10.1
Dibromomethane (1 µg/L)	98.6	8.7
Methyl tert-butyl ether (MTBE) (1 µg/L)	93.1	9.59
Methylene chloride (DCM) (1 µg/L)	100	15.5
Styrene (0.5 µg/L)	96.2	11.5
Tetrahydrofuran (1 µg/L)	96.4	11.9
Trichloromethane (chloroform) (1 µg/L)	96.6	12.5

Section S2: Comparison of Method Reporting Limits (MRLs) with Health-Based Standards

Table S2: Compounds with method reporting limits (MRLs) exceeding their most conservative health-based limits. Comparison of MRLs with the lowest applicable U.S. EPA Maximum Contaminant Levels (MCLs) or Minnesota Department of Health (MDH) Health Risk Limits (HRLs). Only four compounds—vinyl chloride, 1,2-dibromoethane (EDB), 1,2,3-trichloropropane (TCP), and 1,2-dibromo-3-chloropropane (DBCP)—had MRLs slightly above their lowest health-based thresholds, which are among the most stringent regulatory values established nationwide.

Compound	MRL (µg/L)	Lowest Health Limit (µg/L)	Notes
Chloroethene (vinyl chloride)	1	0.2	EPA MCL = 2, but MN chronic HRL = 0.2
1,2-Dibromoethane (EDB)	1	0.03	EPA MCL = 0.05
1,2,3-Trichloropropane (TCP)	1	0.003	MN HRL very low due to carcinogenic risk
1,2-Dibromo-3-chloropropane (DBCP)	1	0.2	EPA MCL = 0.2

Section S3: Evaluation of Sample Preservation, Hold Times and Artefactual Acetone Formation

This section serves as an explanation of the results presented on page 13, referencing the potential causes of acetone formation in our samples, most likely due to high concentrations of dissolved organic carbon reacting with the ascorbic acid preservative over longer hold times, as well as enhanced DBP production over longer hold times. This hypothesis was further explored through laboratory testing to determine the specific reagents and methods that could potentially lead to acetone formation. Further analysis was conducted utilizing older and newer sample preservation methods, as well as different combinations of reagents. The specific methods and sample preservation techniques are detailed below as well. The results of consistent acetone presence in samples was alarming and the documentation of the potential link between reagents, preservation methods, and length of storage before analysis could further support other labs in future work.

The experiment investigated the formation of acetone under different reagent and storage conditions following EPA Methods 524.4 and 524.2, using L-(+)-ascorbic acid, sodium thiosulfate pentahydrate, and hydrochloric acid. Observations showed that ascorbic acid in deionized water produced acetone, while hydrochloric acid alone did not. When combined in tap

water samples, a lower concentration of ascorbic acid and HCl produced less acetone, whereas a higher concentration produced more. Sodium thiosulfate with HCl generated both acetone and iodomethane in deionized and tap water. Lab personnel noted that acetone formation is not due to laboratory contamination but instead arises from reactions involving ascorbic acid and time. Follow-up experiments from lab personnel confirmed that freshly prepared samples showed no acetone, but after 14 days of storage, acetone appeared only in tap samples containing ascorbic acid and HCl, demonstrating that the effect was not due to environmental diffusion. Samples made with older ascorbic acid showed even higher acetone formation after storage, suggesting the age of the reagent influences the reaction. Overall, acetone formation correlates with the presence of organic material, the use and age of the organic quencher (ascorbic acid), and sample storage time, indicating that acetone is likely an artefact of the sample preservation process rather than a constituent originally present in the water.

The results from the extended experiments can be seen in the table below.

Table S3: Results from old and new sample preservation materials, varying sample combinations and days held. *Note that AA means ascorbic acid*

Sample	Date Prepared	Date Analyzed	Days Held	Acetone (ug/L)
Old AA+HCl, DIH2O	2024-04-18	2024-04-18	0	n.a./n.r.
Old AA+HCl, DIH2O	2024-09-23	2024-10-08	15	3.03
Old AA+HCl, Tap	2024-01-11	2024-01-11	0	n.a./n.r.
Old AA+HCl, Tap	2024-09-23	2024-10-08	15	31.05
New AA+HCl, DIH2O	2024-12-30	2024-12-30	0	n.a./n.r.
New AA+HCl, DIH2O	2024-12-30	2025-01-13	14	n.a./n.r.
New AA+HCl, Tap	2024-12-30	2024-12-30	0	n.a./n.r.
New AA+HCl, Tap	2024-12-30	2025-01-13	14	5.89
DIH2O	2024-12-30	2024-12-30	0	n.a./n.r.
DIH2O	2024-12-30	2025-01-13	14	n.a./n.r.
Tap	2024-12-30	2024-12-30	0	n.a./n.r.
Tap	2024-12-30	2025-01-13	14	n.a./n.r.

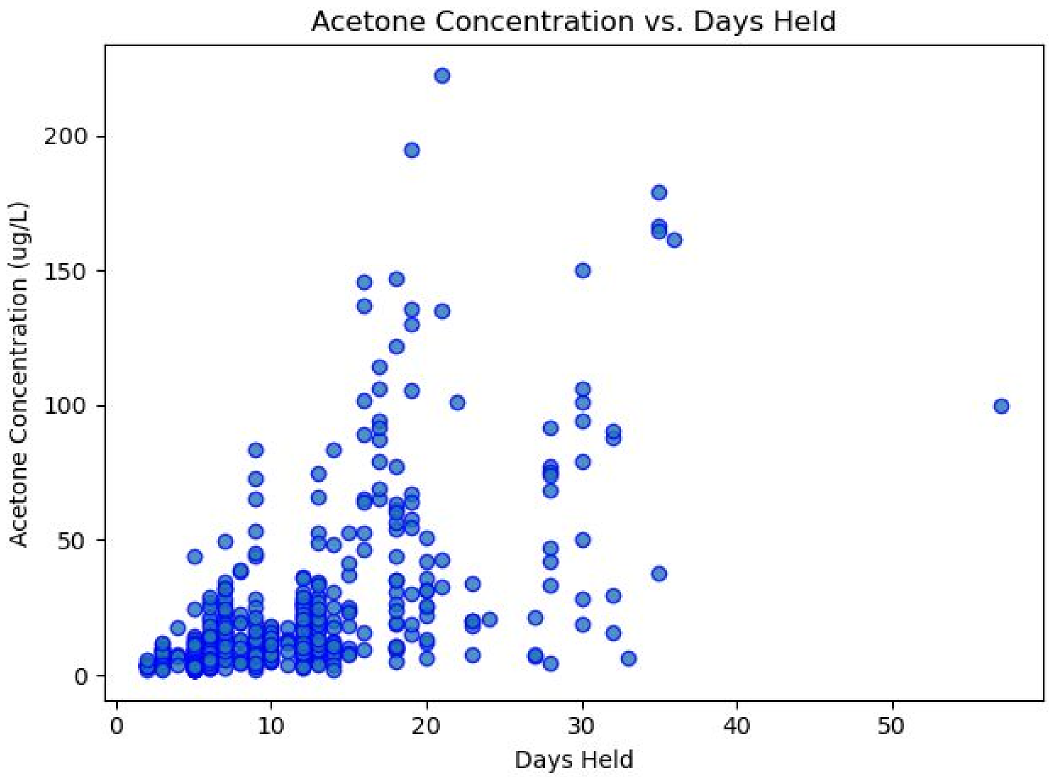

Figure S3-1: Acetone concentration over days held for all samples analyzed throughout the course of the home water sampling program.

Influence of Preservation Methods and Hold Times on VOC and DBP Results:
In addition to investigating acetone artifacts, we also evaluated how sample hold times influenced measured VOC concentrations, particularly for trihalomethanes (i.e. disinfection by-products or DBPs). Early in the sampling campaign, longer storage durations coincided with elevated DBPs, prompting us to examine whether refrigerated storage and preservative chemistry may have contributed to continued DBP formation. To compare hold times meaningfully, we separated samples into non-exceedance and exceedance groups based on whether any measured VOC concentration surpassed its applicable EPA or MDH health-based threshold. This analysis helped elucidate the role of holding times in introducing potential artefacts from sample handling and storage, thereby potentially masking true distribution-system conditions. A sample was classified as an exceedance sample if one or more of the VOCs tested exceeded one of the health limits.

Table S3-2: Summary of sample counts and hold-time statistics for all VOC samples, showing longer mean hold times among exceedance samples compared to non-exceedance samples.

Group	Number of Samples	Mean Hold Time (days)	Std. Dev. (days)
All samples	592	10.41	6.81
Non-exceedance samples	321	8.99	5.57
Exceedance samples	271	12.09	7.7

A histogram illustrating these distributions is provided in Figure S3-2. The differences in hold times between exceedance and non-exceedance samples were evaluated using both parametric and non-parametric tests. Results are summarized below:

Table S3-3. Statistical comparison of hold times between exceedance and non-exceedance samples.

Test	Statistic	p-value	Interpretation
**Welch's t-test**	t = –5.552	< 0.0001	Significant difference in mean hold times
**Mann–Whitney U**	U = 32,953.5	< 0.0001	Significant difference in distributions
**Effect size**	Cohen's d = 0.461	—	Moderate effect

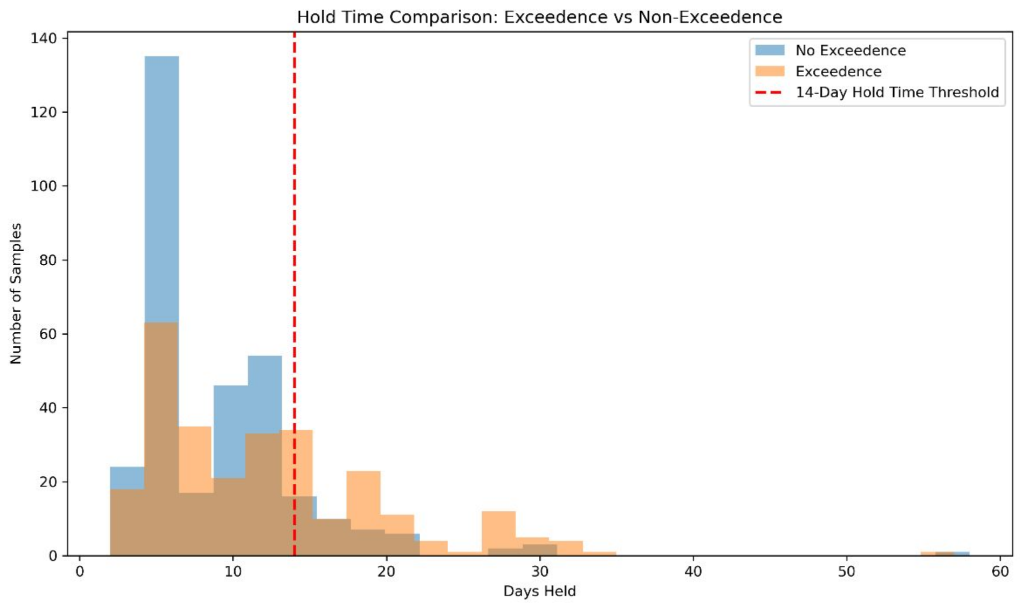

Figure S3-2: Histogram illustrating exceedance distributions as a function of holding time.

Sample Date Also Strongly Influenced Exceedances:
Another interesting pattern that can be seen is that exceedances seemed to be correlated with earlier sampling dates, which may reflect an artifact of the evolving situation, as well as sampling bias towards locations that were accessible following the disaster. Analysis of sampling dates showed that many exceedance samples were collected significantly earlier in the campaign (Figure S3-3). Statistical tests confirmed a strong difference between groups (Welch's *t*-test: $p < 1\times10^{-9}$; Mann–Whitney *U*: $p < 1\times10^{-12}$; Cohen's $d = -0.55$). This pattern reflects the operational context during the initial response period, when GC-MS workflows were still being established, Lahaina remained inaccessible, and most early samples were collected in Kula, an area with higher natural organic carbon that favors DBP formation. Early logistical constraints also produced longer hold times. Together, these factors indicate that the correlation between longer hold times and exceedances was partly driven by the fact that early samples were both more difficult to process rapidly and sourced from water chemistry conditions more prone to trihalomethane formation.

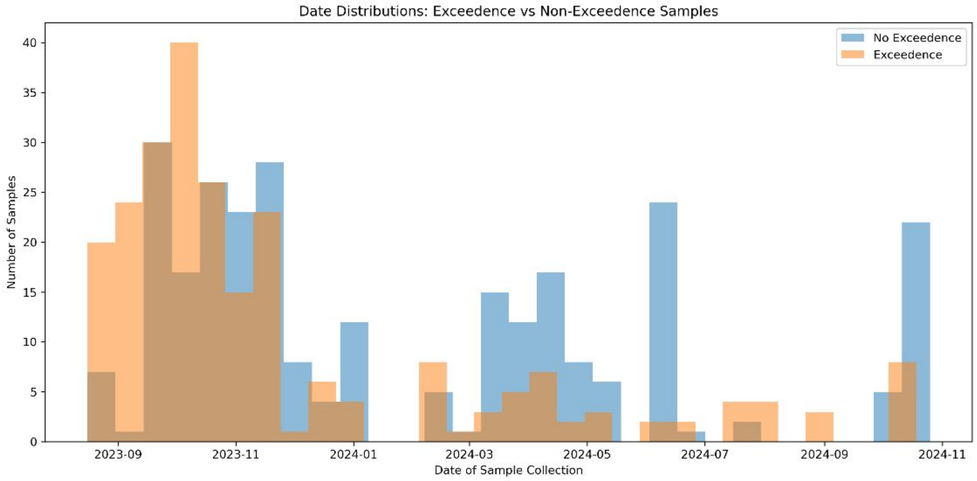

Figure S3-3. Histogram illustrating exceedance distributions as a function of sampling date, showing that many exceedance samples were collected earlier in the sampling campaign.

Across all samples, seven VOCs exceeded one or more health-based thresholds. Nearly all exceedances (684 of 686, or 99.7%) were attributable to regulated disinfection byproducts, primarily trihalomethanes (THMs), including chloroform, bromodichloromethane, dibromochloromethane, bromoform, and total THMs. Only two exceedances involved potential fire-related compounds: chloromethane (methyl chloride), a biomass-burning product detected once in Kula in October 2024, and chloroethene (vinyl chloride), associated with PVC combustion and detected once in Lahaina in December 2023. These rare detections were the only exceedances not linked to DBP formation.

Table S3-4. VOCs with Health-Based Exceedances. VOC's that are not classified as DBPs and may be fire related are shown in **bold.**

Chemical	Number of Exceedances	Notes
Trichloromethane (chloroform)	301	DBP (THM)
Bromodichloromethane	249	DBP (THM)
Bromoform	95	DBP (THM)
Dibromochloromethane	30	DBP (THM)
Total Trihalomethanes (TTHMs)	9	DBP group exceedance
**Chloromethane (methyl chloride)**	**1**	**Potential fire-related; Kula, Oct 2024**
**Chloroethene (vinyl chloride)**	**1**	**Potential fire-related; Lahaina, Dec 2023**

Interpretation:

Although samples with longer hold times exhibited more VOC exceedances, this pattern likely reflects the combined influence of several factors rather than hold time alone. Continued trihalomethane formation during refrigerated storage likely contributed to elevated DBPs, particularly in samples with higher organic carbon. However, early in the sampling campaign, most samples were collected in Kula, an area with naturally elevated organic matter, and GC-MS workflows were still being established, resulting in longer hold times and higher baseline DBPs. These operational and chemical conditions created a sampling bias that magnified the apparent association between hold time and exceedances. Importantly, exceedances attributable to fire-related compounds were rare, with only two isolated detections above health limits, indicating that fire-derived contaminants were largely prevented from entering the distribution system.

Section S4: Analysis of disinfection byproducts (DBP) Pre and Post-Fire:

To evaluate whether the trihalomethanes (TTHMs) detected in post-fire samples were associated with wildfire-related volatile organic compound (VOC) formation or reflected typical disinfectant byproduct processes, we analyzed historical disinfection byproduct (DBP) data for the Lahaina (HI0000214) and Upper Kula (HI0000215) drinking water systems. These data were obtained from the Maui Department of Water Supply and include results from both wells and distribution-system monitoring sites. Specifically, quarterly DBP measurements spanning January 2018 through 2025 were requested and compiled for both systems. The datasets include the four individual compounds that constitute total trihalomethanes (TTHMs): chloroform, bromodichloromethane, dibromochloromethane, and bromoform, as well as haloacetic acid species (HAA5). We have made these data publicly available in the project's GitHub repository: (Dynamic:
https://github.com/cshuler/VOC_Processing_Maui/tree/main/Paper_Resources/Data,
Permanent: https://doi.org/10.5281/zenodo.17365218)
214 – Lahaina DBP Data 2018 – 2025.csv and 215 – Upper Kula DBP Data 2018 – 2025.csv

Results of Analysis: From 2018–2022 (pre-fire), TTHM concentrations in both systems fluctuated within typical regulatory ranges. The maximum recorded pre-fire value was 73 µg/L (Figure S4) in the Lahaina distribution system (July 2023) and 55 µg/L in the Kula system (October 2020). Comparing 2022 maxima to our post-fire residential sampling program, 36 of our 183 Kula samples and 28 of our 127 Lahaina samples exceeded the 2022 thresholds of 44 µg/L and 62 µg/L, respectively. Across both systems, the Hawaii Department of Health (HDOH) compliance testing did not report TTHM concentrations above the EPA Maximum Contaminant Level (MCL) of 80 µg/L. However, several DBPs exceeded Minnesota Department of Health (MDH) health-based guidance values during the study period. These included Dibromochloromethane (MDH sub-chronic value = 10 µg/L) was exceeded four times in Lahaina—October 7 2022 (two samples), July 3 2023, and October 30 2023—with a maximum of 21 µg/L. These exceedances consistently occurred in late summer to early fall, suggesting a seasonal pattern. Chloroform (trichloromethane) (MDH chronic value = 20 µg/L) showed more frequent exceedances: 18 in Kula (maximum = 47 µg/L) and 18 in Lahaina (maximum = 26 µg/L). Exceedances typically occurred during July–October in multiple years, consistent with warmer temperatures or operational factors that may promote DBP formation. Bromodichloromethane (MDH = 30 µg/L) and bromoform showed no recorded exceedances.

Interpretation: This analysis demonstrates that DBPs were present at measurable concentrations in both systems before and after the fires, and that periodic exceedances of health-based guidance values occur under normal operating conditions. Post-fire TTHM values in our residential samples were generally higher than utility-reported values, likely reflecting differences in sampling locations and water age. Our samples were drawn from household service lines rather than hydrant or main-line compliance sites. Extended residence times and increased chlorination during system restoration could therefore account for higher TTHM levels, overlapping with possible minor fire-related effects.

Collectively, these results indicate that elevated DBPs following the Maui wildfires are best explained by a combination of operational factors, 1) water residence time in service lines, 2) potential localized fire impacts on distribution materials, and 3) operational shifts in response to the fire's impact on the water systems. We do not find any evidence to indicate that an increase in organic matter typical of surface-water post-fire systems, especially in light of the fact that Maui DWS was clear in all messaging there they did not suffer any fire-related impacts to source waters or watersheds, as the 2023 fires primarily affected residential and urban areas rather than forested catchments.

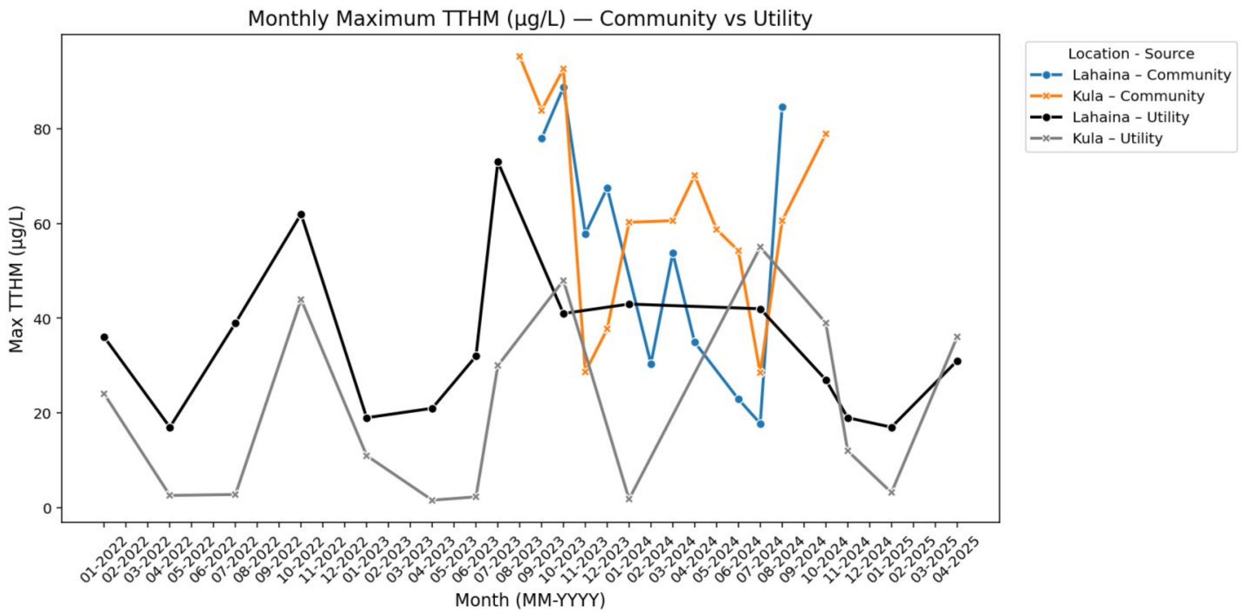

Figure S4-1: Per month maximums of chemicals classified as total trihalomethanes for both Lahaina and Kula samples through the course of the sampling program and distribution systems and wells in the Kula and Lahaina systems (2022-2025).

Section S5: Supplementary Figures

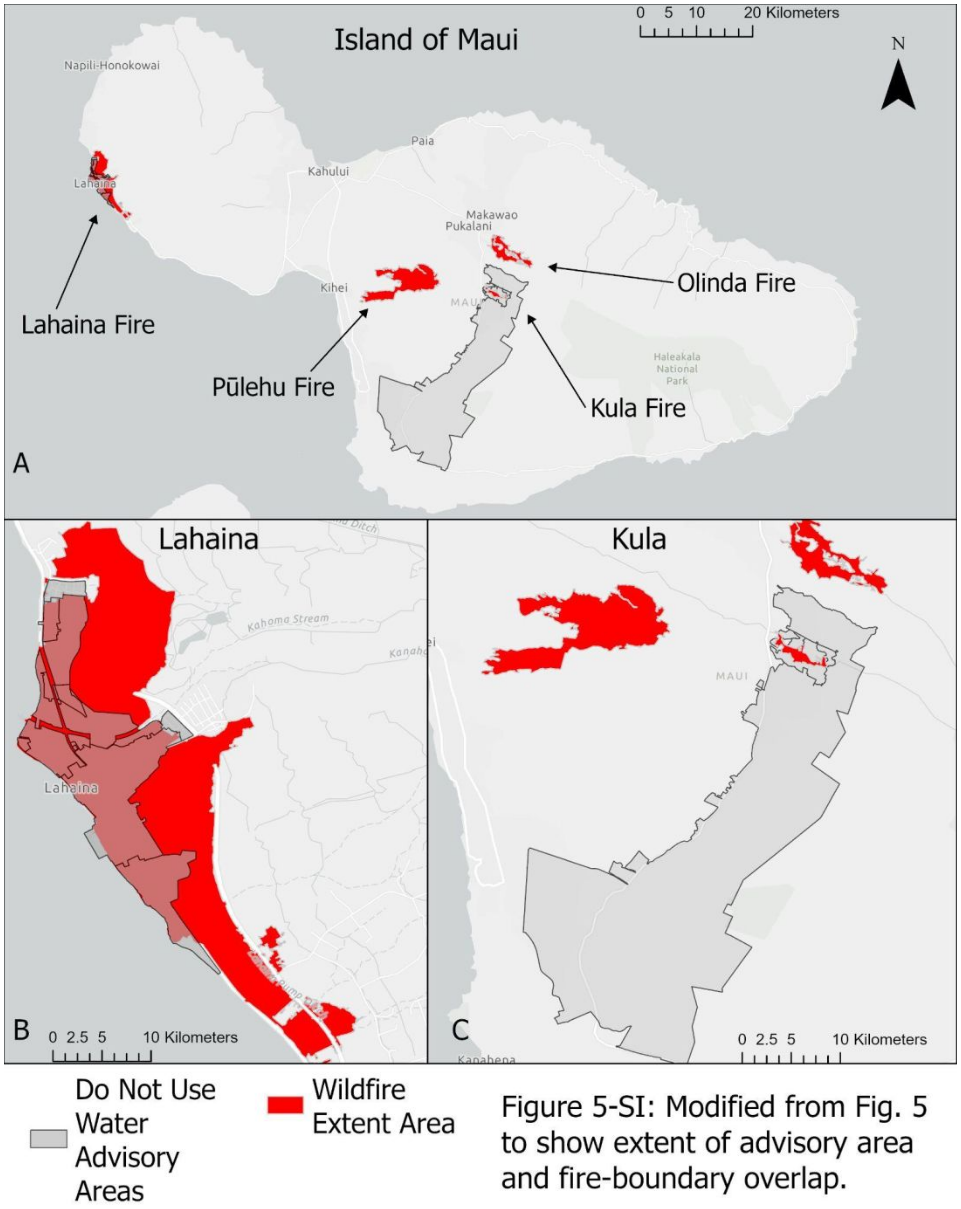

Figure S5-1: Modified from Figure 5, titled Figure 5-SI - Intended to show the extent of advisory area and fire-boundary overlap. This supplementary figure highlights the relationship between the post-fire water advisory areas and wildfire extent in Lahaina. The advisory areas are outlined with semi-transparent grey to allow visibility of the underlying fire-affected regions (shown in red). This visualization was created to clarify spatial overlap that could not be effectively represented in the main Figure 5 due to the density of sample locations and multiple overlaid datasets.

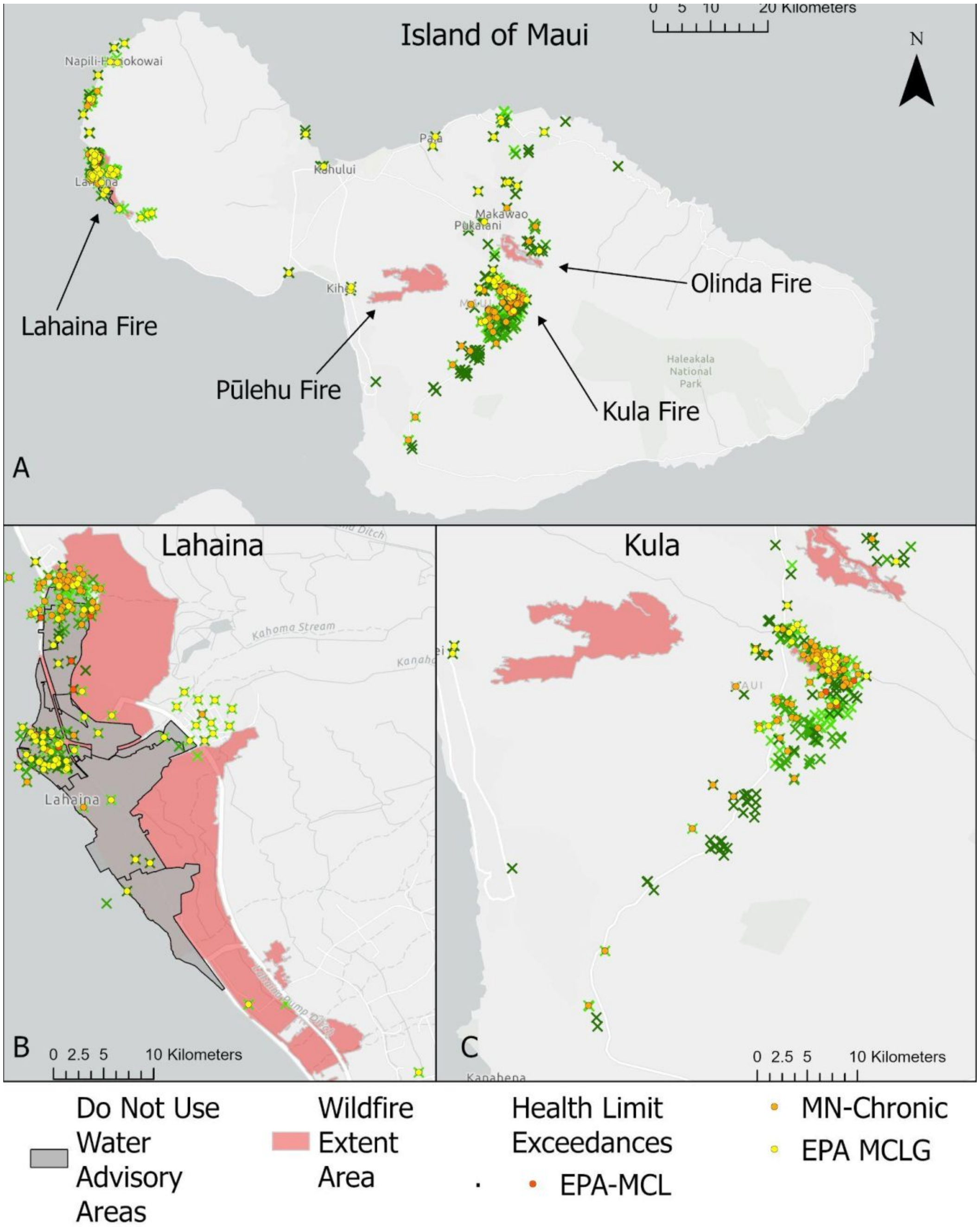

Figure S5-2: Locations of water samples on Maui where one or more volatile organic compounds (VOCs) exceeded health-based standards. Points are colored by exceedance category: red indicates samples exceeding an EPA Maximum Contaminant Level (MCL), orange indicates samples exceeding a Minnesota chronic exposure limit but not an MCL, and yellow indicates samples exceeding an EPA Maximum Contaminant Level Goal (MCLG) only. Fire perimeters from the August 2023 wildfires and “Do Not Use Water” advisory areas are shown for context. This figure supplements Table 1 and directly illustrates the spatial distribution of exceedances across affected areas.

Number of Chemicals Detects Above Any Health Limit in Kula

Total number of samples: 183

Chemical	Number of Detects	MDH Chronic	MDH Sub-Chronic	EPA MCLG	EPA MCL
Chloromethane (methyl chloride)	1	-	-	-	0
Chloroethene (vinyl chloride)	0	0	-	0	0
Bromomethane (methyl bromide)	0	0	-	-	-
Chloroethane (ethyl chloride)	0	-	-	-	-
Trichlorofluoromethane	0	0	-	-	-
Diethyl ether	0	-	-	-	-
1,1-Dichloroethene	0	-	-	-	-
Acetone	163	0	0	-	-
Iodomethane	0	-	-	-	-
Carbon disulfide	4	0	-	-	-
3-Chloropropene (allyl chloride)	0	0	-	-	-
Methylene chloride (DCM)	11	0	-	-	-
trans-1,2-Dichloroethene	0	0	-	0	-
Methyl tert-butyl ether (MTBE)	2	0	0	-	-
1,1-Dichloroethane	0	0	0	0	-
2,2-Dichloropropane	0	-	-	0	-
cis-1,2-Dichloroethene	0	0	0	0	-
2-Butanone (MEK)	13	0	-	-	-
Methyl acrylate	0	-	-	-	-
Methacrylonitrile	0	-	-	-	-
Bromochloromethane	0	-	-	-	-
Tetrahydrofuran	21	0	0	-	-
Trichloromethane (chloroform)	179	79	79	4	-
1,1,1-Trichloroethane	0	0	0	0	0
1-Chlorobutane (butyl chloride)	0	-	-	-	-
Carbon tetrachloride	0	0	0	0	0
1,1-Dichloropropene	0	-	-	-	-
Benzene	183	0	0	0	0
1,2-Dichloroethane	0	0	0	0	0
Trichloroethene	0	-	-	-	-
1,2-Dichloropropane	0	0	0	0	0
Dibromomethane	0	-	-	-	-
Methyl methacrylate	0	-	-	-	-
Bromodichloromethane	99	0	0	99	-
2-Nitropropane	0	-	-	-	-
cis-1,3-Dichloropropene	0	-	-	-	-
4-Methyl-2-pentanone (MIBK)	0	-	-	-	-
Toluene	0	0	0	0	0
trans-1,3-Dichloropropene	0	-	-	-	-
Ethyl methacrylate	0	-	-	-	-
1,1,2-Trichloroethane	0	0	-	-	0
Tetrachloroethene	0	-	-	-	-
1,3-Dichloropropane	0	-	-	-	-
2-Hexanone	0	-	-	-	-
Dibromochloromethane	59	2	-	0	-
1,2-Dibromoethane (EDB)	0	0	0	-	-
Chlorobenzene	0	0	-	0	0
1,1,1,2-Tetrachloroethane	0	0	-	-	-
Ethylbenzene	0	0	0	0	0
m/p-Xylene	0	0	0	0	0
o-Xylene	0	0	0	0	0
Styrene	0	-	-	0	0
Bromoform	4	-	-	4	-
Isopropylbenzene (cumene)	0	0	-	-	-
Bromobenzene	0	-	-	-	-
1,1,2,2-Tetrachloroethane	0	-	-	-	-
1,2,3-Trichloropropane (TCP)	0	0	0	-	-
trans-1,4-Dichloro-2-butene	0	-	-	-	-
n-Propylbenzene	0	-	-	-	-
2-Chlorotoluene	0	-	-	-	-
1,3,5-Trimethylbenzene	0	0	0	-	-
4-Chlorotoluene	0	-	-	-	-
tert-Butylbenzene	0	-	-	-	-
Pentachloroethane	0	-	-	-	-
1,2,4-Trimethylbenzene	0	0	0	-	-
1-Methylpropylbenzene (sec-butylbenzene)	0	-	-	-	-
1,3-Dichlorobenzene	0	-	-	-	-
4-Isopropyltoluene (p-cymene)	0	-	-	-	-
1,4-Dichlorobenzene	0	0	0	-	-
n-Butylbenzene	0	-	-	-	-
1,2-Dichlorobenzene	0	0	-	-	-
Hexachloroethane	0	-	-	-	-
1,2-Dibromo-3-chloropropane (DBCP)	0	-	0	0	0
Nitrobenzene	0	-	-	-	-
1,2,4-Trichlorobenzene	0	0	0	0	0
Hexachloro-1,3-butadiene	0	-	-	-	-
Naphthalene	0	0	0	-	-
1,2,3-Trichlorobenzene	0	-	-	-	-
Total Trihalomethanes	180	-	-	-	4

Figure S5-3. Figure of detects and comparison to various health limits for Kula. This specific table was created and designed for the Information Hub to provide clear, location specific results. Note: This a direct screenshot from the Information Hub

Number of Chemicals Detects Above Any Health Limit in Lahaina

Total number of samples: 127

Chemical	Number of Detects	MDH Chronic	MDH Sub-Chronic	EPA MCLG	EPA MCL
Chloromethane (methyl chloride)	0	-	-	-	0
Chloroethene (vinyl chloride)	1	0	-	1	0
Bromomethane (methyl bromide)	0	0	-	-	-
Chloroethane (ethyl chloride)	0	-	-	-	-
Trichlorofluoromethane	0	0	-	-	-
Diethyl ether	0	-	-	-	-
1,1-Dichloroethene	0	-	-	-	-
Acetone	120	0	0	-	-
Iodomethane	0	-	-	-	-
Carbon disulfide	0	0	-	-	-
3-Chloropropene (allyl chloride)	0	0	-	-	-
Methylene chloride (DCM)	0	0	-	-	-
trans-1,2-Dichloroethene	0	0	-	0	-
Methyl tert-butyl ether (MTBE)	0	0	0	-	-
1,1-Dichloroethane	0	0	0	0	-
2,2-Dichloropropane	0	-	-	0	-
cis-1,2-Dichloroethene	0	0	0	0	-
2-Butanone (MEK)	13	0	-	-	-
Methyl acrylate	0	-	-	-	-
Methacrylonitrile	0	-	-	-	-
Bromochloromethane	1	-	-	-	-
Tetrahydrofuran	6	0	0	-	-
Trichloromethane (chloroform)	124	52	52	0	-
1,1,1-Trichloroethane	0	0	0	0	0
1-Chlorobutane (butyl chloride)	0	-	-	-	-
Carbon tetrachloride	0	0	0	0	0
1,1-Dichloropropene	0	-	-	-	-
Benzene	0	0	0	0	0
1,2-Dichloroethane	0	0	0	0	0
Trichloroethene	0	-	-	-	-
1,2-Dichloropropane	0	0	0	0	0
Dibromomethane	5	-	-	-	-
Methyl methacrylate	0	-	-	-	-
Bromodichloromethane	120	0	0	120	-
2-Nitropropane	0	-	-	-	-
cis-1,3-Dichloropropene	0	-	-	-	-
4-Methyl-2-pentanone (MIBK)	0	-	-	-	-
Toluene	0	0	0	0	0
trans-1,3-Dichloropropene	0	-	-	-	-
Ethyl methacrylate	0	-	-	-	-
1,1,2-Trichloroethane	0	0	-	-	0
Tetrachloroethene	0	-	-	-	-
1,3-Dichloropropane	0	-	-	-	-
2-Hexanone	0	-	-	-	-
Dibromochloromethane	119	26	-	0	-
1,2-Dibromoethane (EDB)	0	0	0	-	-
Chlorobenzene	0	0	-	0	0
1,1,1,2-Tetrachloroethane	0	0	-	-	-
Ethylbenzene	0	0	0	0	0
m/p-Xylene	0	0	0	0	0
o-Xylene	0	0	0	0	0
Styrene	0	-	-	0	0
Bromoform	61	-	-	61	-
Isopropylbenzene (cumene)	0	0	-	-	-
Bromobenzene	0	-	-	-	-
1,1,2,2-Tetrachloroethane	0	-	-	-	-
1,2,3-Trichloropropane (TCP)	0	0	0	-	-
trans-1,4-Dichloro-2-butene	0	-	-	-	-
n-Propylbenzene	0	-	-	-	-
2-Chlorotoluene	0	-	-	-	-
1,3,5-Trimethylbenzene	0	0	0	-	-
4-Chlorotoluene	0	-	-	-	-
tert-Butylbenzene	0	-	-	-	-
Pentachloroethane	0	-	-	-	-
1,2,4-Trimethylbenzene	0	0	0	-	-
1-Methylpropylbenzene (sec-butylbenzene)	0	-	-	-	-
1,3-Dichlorobenzene	0	-	-	-	-
4-Isopropyltoluene (p-cymene)	0	-	-	-	-
1,4-Dichlorobenzene	0	0	0	-	-
n-Butylbenzene	0	-	-	-	-
1,2-Dichlorobenzene	0	0	-	-	-
Hexachloroethane	0	-	-	-	-
1,2-Dibromo-3-chloropropane (DBCP)	0	-	0	0	0
Nitrobenzene	0	-	-	-	-
1,2,4-Trichlorobenzene	0	0	0	0	0
Hexachloro-1,3-butadiene	0	-	-	-	-
Naphthalene	0	0	0	-	-
1,2,3-Trichlorobenzene	0	-	-	-	-
Total Trihalomethanes	126	-	-	-	5

Figure S5-4: Figure of detects and comparison to various health limits for Lahaina. This specific table was created and designed for the Information Hub to provide clear, location specific results. Note: This a direct screenshot from the Information Hub

Section S6: Maui Post-Fire Community Drinking-Water Information Hub Archive: Website Output and Visualization

The following figures provide supporting visual materials referenced in the main text. The figures below are screenshots taken directly from the Maui Post-Fire Community Drinking-Water Information Hub (originally located at: https://www.wrrc.hawaii.edu/maui-post-fire-community-water-info-hub/,
and now permanently archived at
https://web.archive.org/web/20250917032434/https://www.wrrc.hawaii.edu/maui-post-fire-community-water-info-hub/), showing how residents accessed and interpreted results for Kula and Lahaina sampling locations. The latter figure provide an additional view into the web-app based visualization tool we used to display the sample results in spatial form.

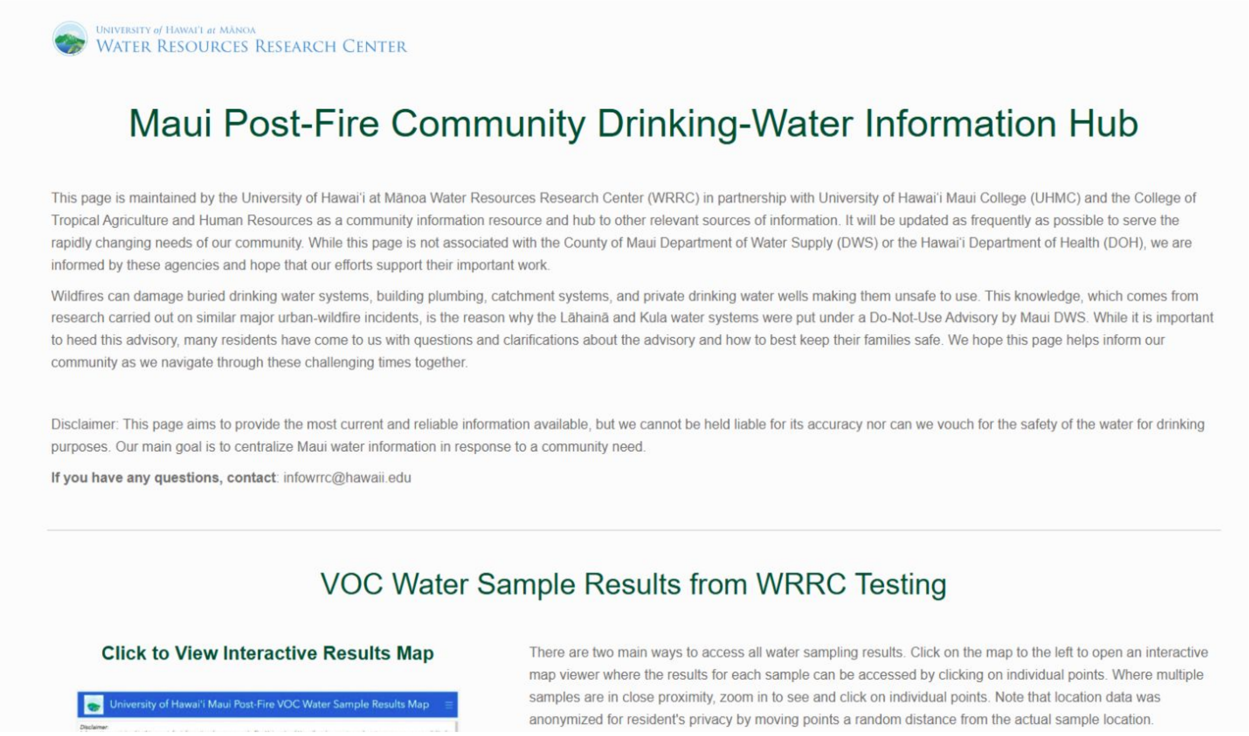

Figure S6-1: The home page for the Info Hub

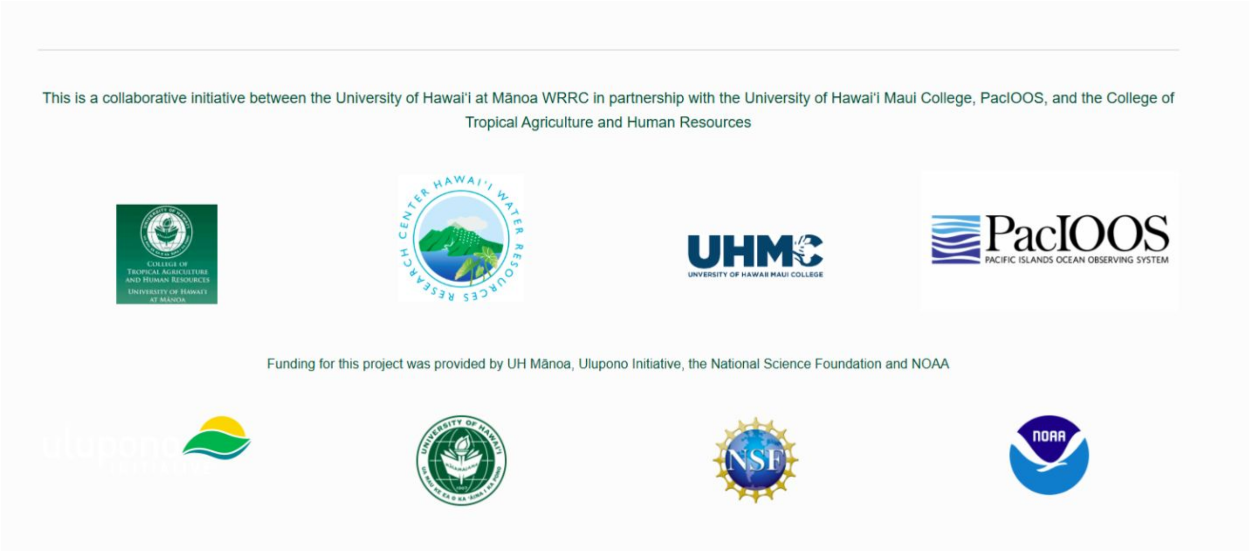

Figure S6-2: The acknowledgements page for the Info Hub

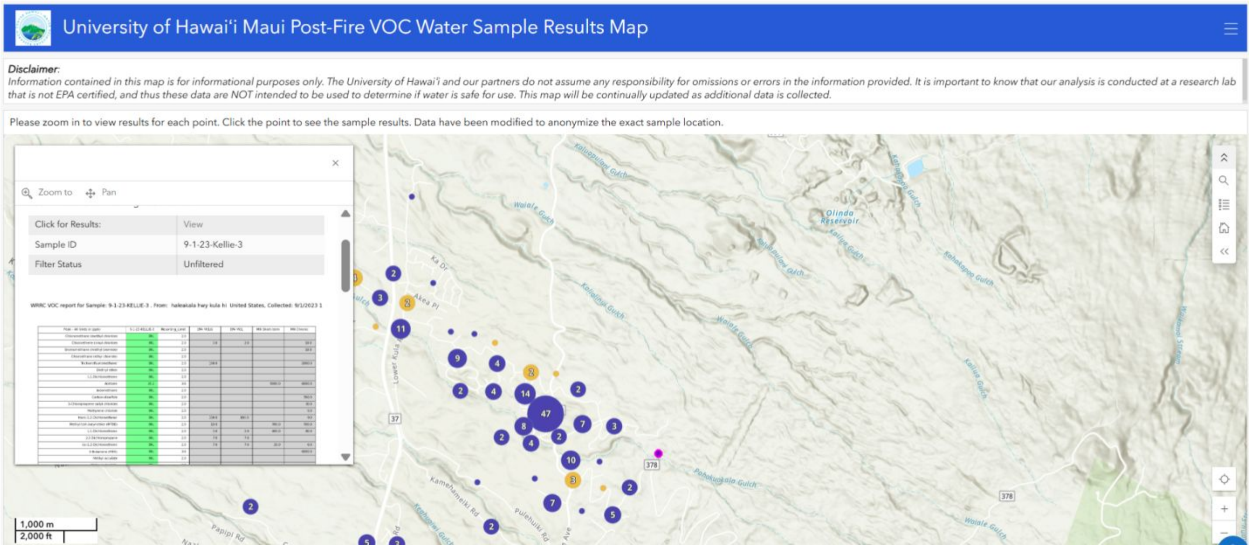

Figure S6-3: The spatial map-based web-app we developed for users to see their sample results in a spatial format.
